# Complete Genome Assembly of *Amycolatopsis bartoniae* DSM 45807^T^ Allows the Characterization of a Novel Glycopeptide Biosynthetic Gene Cluster

**DOI:** 10.3390/genes15121651

**Published:** 2024-12-22

**Authors:** Anastasia Stepanyshyn, Christian Rückert-Reed, Tobias Busche, Bohdan Yaruta, Andres Andreo-Vidal, Flavia Marinelli, Jörn Kalinowski, Oleksandr Yushchuk

**Affiliations:** 1 Department of Genetics and Biotechnology, Ivan Franko National University of Lviv, 79005 Lviv, Ukraine; nastia.stepanyshyn@gmail.com (A.S.); 3bogdanyaryta3@gmail.com (B.Y.); 2 Technology Platform Genomics, CeBiTec, Bielefeld University, Sequenz 1, 33615 Bielefeld, Germany; christian.rueckert@uni-bielefeld.de (C.R.-R.); tobias.busche@uni-bielefeld.de (T.B.); joern@cebitec.uni-bielefeld.de (J.K.); 3 Medical School OWL, Bielefeld University, Sequenz 1, 33615 Bielefeld, Germany; 4 Department of Biotechnology and Life Sciences, University of Insubria, 21100 Varese, Italy; andresandreoiv@gmail.com (A.A.-V.); flavia.marinelli@uninsubria.it (F.M.)

**Keywords:** genome sequencing, biosynthetic gene cluster, glycopeptide antibiotics, in silico analysis, actinomycetes

## Abstract

Background: Glycopeptide antibiotics (GPAs) are a very successful class of clinically relevant antibacterials, used to treat severe infections caused by Gram-positive pathogens, e.g., multidrug resistant and methicillin-resistant staphylococci. The biosynthesis of GPAs is coded within large biosynthetic gene clusters (BGCs). In recent years, modern DNA sequencing technologies have allowed the identification and characterization of multiple novel GPA BGCs, leading to the discovery of novel compounds. Our previous research anticipated that the genome of *Amycolatopsis bartoniae* DSM 45807^T^ carries a novel GPA BGC, although the genomic sequence quality available at that time did not allow us to characterize its organization properly. Objectives: To address this gap, in the current work we aimed to produce a complete genome assembly of *A. bartoniae* DSM 45807, and to identify and analyze the corresponding GPA BGC. Methods: Bioinformatic and microbiological methods were utilized in this research. Results: We de novo sequenced and completely assembled the genome of *A. bartoniae* DSM 45807, and fully characterized the BGC of interest, named *aba*. This BGC has an unusual gene organization and it contains four genes for sulfotransferases, which are considered to be rare in GPA BGCs. Our pathway prediction indicated that *aba* encodes the biosynthesis of a putatively novel GPA, although we were not able to detect any GPA production under different cultivation conditions, implying that *aba* pathway is inactive. Conclusions: Our results indicate *aba* as a promising source for new GPA tailoring enzymes.

## 1. Introduction

Glycopeptide antibiotics (GPAs) are a class of clinically successful lipid II binders [1], produced by various bacterial genera within the phylum *Actinomycetota* [2]. GPAs are non-ribosomal peptides synthesized through multi-step biosynthetic pathways [3]; the corresponding genes are co-localized, forming large biosynthetic gene clusters (BGCs) [4,5,6]. By binding to the d-Ala-d-Ala termini of nascent peptidoglycan [7], GPAs inhibit upstream transglycosylation and transpeptidation reactions and are highly effective against Gram-positive bacteria, including methicillin-resistant staphylococci and enterococci [8].

Currently, five GPAs are in clinical use: the natural compounds vancomycin (produced by various *Amycolatopsis* spp. [9]) and teicoplanin (produced by *Actinoplanes teichomyceticus* ATCC 31121 [6]), as well as the semi-synthetic compounds oritavancin, telavancin, and dalbavancin [10], derived, respectively, from chloroeremomycin (produced by *Kibdelosporangium aridum* A82846 [11]), vancomycin, and A40926 (produced by *Nonomuraea gerenzanensis* ATCC 39727 [12]). However, the chemical diversity of GPAs extends far beyond these clinically relevant compounds, and all known GPAs to date are divided into five classes based on their chemical structures [2]. Notably, types I-IV (also known as dalbaheptides [13]) are lipid II binders, while type V compounds inhibit cell wall turnover [14]. Due to the lack of glycosylations and other structural features, type V compounds were recently proposed to be referred to as GRPs—glycopeptide-related peptides [15].

Thanks to the advances in genome sequencing during the genomic and post-genomic eras, the chemical diversity of GPAs has been rivaled by the genetic diversity of GPA BGCs. A significant portion of this diversity comes from uninvestigated bacteria, and it remains unclear which compounds, if any, are produced by some of these clusters. Unfortunately, vancomycin- and teicoplanin-resistant pathogens continue to spread [16,17], and reports of resistance to semi-synthetic GPAs are also emerging [18]. Thus, it is crucial to investigate the natural diversity of GPA BGCs to identify novel GPA producers and to understand the variety of non-ribosomal peptide synthetase (NRPS) and tailoring genes, which could be harnessed in the combinatorial biosynthesis of new GPAs.

In recent years, several excellent comparative bioinformatics studies of GPA BGCs have been conducted, providing detailed insights into GPA evolution [15,19,20,21] and enabling de novo engineering of entire GPA biosynthetic pathways [21]. In one of our previous studies, focusing on the distribution and evolution of GPA-resistance genes across various *Actinomycetota* spp. [22], we identified a novel GPA BGC in the genome of *Amycolatopsis bartoniae* CGMCC 4.7679 (=DSM 45807). At the time, we noted its unusual genetic organization in comparison with other GPA BGCs from *Amycolatopsis* spp. [5,23,24]. However, the low quality of the available genome draft, in which the BGC-related genes were located on multiple contigs, prevented us from drawing unambiguous conclusions about its organization. This likely explains why subsequent studies on the evolution of GPA BGCs omitted the analysis of the BGC from *A. bartoniae* CGMCC 4.7679 as well [15,21].

To address this gap, in the current work we produced a complete genome assembly of *A. bartoniae* DSM 45807, enabling us to identify and analyze the corresponding GPA BGC, which we have named *aba* (derived from the ***A**mycolatopsis **ba**rtoniae* taxon name), and to predict its biosynthetic pathway. We also discovered that *aba* contains a set of unusual genes, whose products may be involved in the tailoring reactions of GPA biosynthesis but have not been previously reported in GPA BGCs and studied experimentally. Furthermore, we identified orthologues of one such gene (a GT1-family glycosyltransferase) in a set of previously unknown GPA BGCs. Finally, we tested the growth and antimicrobial properties of *A. bartoniae* DSM 45807 under various laboratory conditions, revealing that the GPA biosynthetic pathway is inactive. Our findings contribute to the understanding of tailoring genes in GPA biosynthesis and outline new avenues for experimental investigations into novel GPA production in *A. bartoniae* DSM 45807.

## 2. Materials and Methods

### 2.1. Sequencing and Assembly of A. bartoniae DSM 45807 Genome

*A. bartoniae* DSM 45807^T^ was obtained from Deutsche Sammlung von Mikroorganismen und Zellkulturen (DSMZ). Genomic DNA of strain *A. bartoniae* DSM 45807 was isolated using the NucleoSpin^®^ Microbial DNA kit (MACHEREY-NAGEL GmbH & Co. KG, Düren, Nordrhein-Westfalen, Germany). The genome was assembled using a combination of MiSeq Illumina and GridION Oxford Nanopore Technologies (ONT) data. For library preparation, the TruSeq DNA PCR-free high-throughput library prep kit (Illumina, San Diego, CA, USA) and the SQK-LSK109 ligation sequencing kit (ONT, Oxford, Oxfordshire, UK) were used with native barcoding without prior shearing of the DNA. To generate the short reads, a 2 ×  300-nucleotide run (MiSeq reagent kit v3, Illumina, 600 cycles) was executed. Reads were trimmed and filtered using trimmomatic [25] PE with option -validatePairs and trimmers SLIDINGWINDOW:5:10, CROP:300, and MINLEN:250. The long reads were generated on a GridION platform using a R9.4.1 flow cell. Base calling and demultiplexing were performed using guppy v6.1.2 with model DNA_r9.4.1_450bps_sup. Reads were then trimmed with cutadapt [26] with parameters -e 0.2 --trimmed-only, and -g AAGGTTAANNNNNNNNNNNNNNNNNNNNNNNNCAGCACCT followed by a second invocation of CUTADAPT with parameters -e 0.2, -m 1000, and -a AGGTGCTGNNNNNNNNNNNNNNNNNNNNNNNNTTAACCTTAGCAAT.

The trimmed data are available from SRA via BioProject PRJNA1184163. From the trimmed long reads, the assembly was generated using flye v.2.9 [27]. The resulting contig was polished with pilon v1.22 [28] using bowtie2 [29] for mapping. The resulting contig representing the circular genome was annotated using the PGAP pipeline [30,31]. The annotated chromosome is available under the GenBank accession number CP174150.

### 2.2. In Silico Analysis of Nucleic Acid and Amino Acid Sequences from A. bartoniae DSM 45807 GPA BGC

Geneious Prime 2025.0.3 was used for the routine analysis of nucleic and amino acid sequences. Conserved domains were identified using CD-Search [32]. mega (v.11.0.13) [33] was used for the multiple amino acid sequence alignment and phylogenetic reconstructions. BGCs were predicted using antiSMASH 7.0. NRPS non-ribosomal codes were obtained from antiSMASH-incorporated NRPSpredictor 2 [34].

### 2.3. Media and Conditions for A. bartoniae DSM 45807 Cultivation

*A. bartoniae* DSM 45807 was routinely cultivated on ISP5 agar at 30 °C. Spore suspensions were prepared from 168-h ISP5 lawns as previously described [35]. For genomic DNA isolation, *A. bartoniae* DSM 45807 was cultivated in liquid ISP2 medium for up to 96 h on an orbital shaker at 220 rpm, 30 °C.

To evaluate the antimicrobial activities of *A. bartoniae* DSM 45807 the following agar media were used: MM, TM1, Sabouraud dextrose agar (SDA, CONDALAB, Madrid, Spain), soil extract agar (SEA), MYM, potato dextrose agar (PDA, CONDALAB), YMPG, Czapek agar (CzA), R5, and ISP1-7.

To test the biomass accumulation and antimicrobial activities of the submerged cultures of *A. bartoniae* DSM 45807, the following liquid media were used: SEED, tryptic soy broth (TSB, CONDALAB), YMPG, E25, E26, VSP, GYM, R5, TM1, FM2, and ISP2. Unless otherwise stated, all media components were sourced from Sigma–Aldrich (USA, St. Louis, MO, USA). Compositions of all media and corresponding references are given in the Electronic Supplementary Material.

To investigate biomass accumulation, submerged cultures of *A. bartoniae* DSM 45807 were started in 250-mL Erlenmeyer flasks containing 30 mL of SEED, TSB, YMPG, E25, E26, VSP, and VSP without saccharose, or GYM medium added with 12 (⌀0.5 mm) glass beads. Each flask was inoculated with approximately 10⁷ spores and incubated on an orbital shaker at 220 rpm and 30 °C for up to 72 h. At 24, 48, and 72-h time points, 1 mL of broth was sampled for growth inhibition assays. At 72 h, 20 mL of broth was collected, and biomass was separated by centrifugation (15 min, 6000 rcf), washed with deionized water, and weighed to determine fresh biomass weight. Samples were then incubated at 60 °C for 16 h to obtain dry weight.

To test GPA production, precultures of *A. bartoniae* DSM 45807 were grown in SEED medium under the same conditions described above. After 72 h, 2.5% (*v*/*v*) of the SEED preculture was transferred into 250-mL Erlenmeyer flasks containing 30 mL of R5, TM1, FM2, or ISP2 medium, along with 12 (⌀0.5 mm) glass beads. These cultures were incubated for up to 168 h on an orbital shaker at 220 rpm and 30 °C. At 24, 48, 72, 96, 120, 144, and 168-h time points, 1 mL of broth was collected for growth inhibition assays.

### 2.4. Agar Plug and Kirby-Bauer Disc Diffusion Assays

An agar plug antibiotic diffusion assay was used to detect the antimicrobial activities of *A. bartoniae* DSM 45807 cultivated on various agar media (see above). Agar plugs (⌀5 mm) were cut from *A*. *bartoniae* DSM 45807 lawns and tested for growth inhibition against the following cultures: *Escherichia coli* DH5α [36] cultured on LB agar [36] (2  ×  10^7^ *E. coli* cells per 50 mL of medium); *Debaryomyces hansenii* VKM Y-9 (*Ascomycota*) [37] cultured on TSB agar (2  ×  10^6^ cells per 50 mL of medium); *Bacillus subtilis* HB0950 [38,39] cultured on Mueller Hinton agar (MHA, CONDALAB) supplemented with 25 µg/mL of X-Gal (Thermo Fisher Scientific, Waltham, MA, USA) (2  ×  10^7^ spores per 50 mL of medium). *B. subtilis* HB0950 is a reporter strain containing a *lacZ* gene (coding for β-galactosidase) fused to the *liaI* promoter (*pliaI*), which activates *lacZ* expression in response to cell wall stress caused by lipid II binders (including GPAs) [38,39]. When *B. subtilis* HB0950 is cultured in the presence of X-Gal, GPA production induces the chromogenic conversion of X-Gal at the margins of growth inhibition zones.

For Kirby–Bauer disc diffusion assays, ⌀5 mm Whatman paper discs were soaked with 100 µL of *A. bartoniae* DSM 45807 culture supernatants and placed on plates containing spores of *B. subtilis* HB0950 (prepared as described above).

The assay plates were incubated at 37 °C (30 °C for *D. hansenii* VKM Y-9) and analyzed after 20 h.

### 2.5. Scanning Electron Microscopy

For SEM, *A. bartoniae* DSM 45807 lawns were cultivated for 120 h on ISP5 agar. Thin slices of the lawn surfaces were cut, coated with thin layers of silver *in vacuo*, and imaged with JSM-T220a SEM (JEOL, Tokyo, Japan) using a 25 kV electron beam.

## 3. Results

### 3.1. Characterization of the GPA BGC from A. bartoniae DSM 45807 and Biosynthetic Pathway Prediction

The genome of *A. bartoniae* DSM 45,807 consists of a single circular chromosome of 7.836 Mbp, with a GC content of 72.5%. No plasmids were identified. The genome was predicted to contain 7323 CDSs, 3 rRNA operons, and 49 tRNA genes. The 16S rRNA genes are microheterogeneous, with one gene (*AMYBAR_002252*) differing by 9 bp mismatches from the other two (Figure S1).

AntiSMASH [34] analysis identified 29 regions resembling specialized metabolites-coding BGCs, 20 of which showed significant similarity to known BGCs or contained recognizable core biosynthetic genes, including the one for a GPA BGC (Table S1).

**Figure 1:**
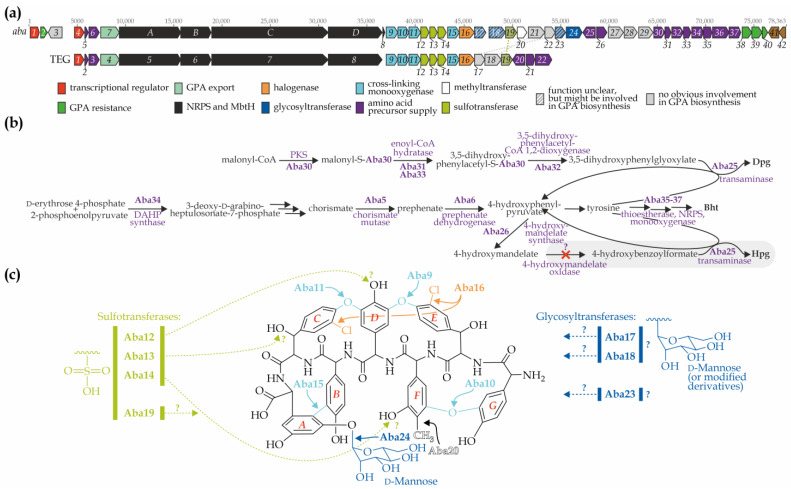
Organization of the *aba* BGC from *A. bartoniae* DSM 45807 and prediction of the encoded biosynthetic pathway: (**a**) genetic organization of the *aba* BGC comparing to that of the TEG BGC, obtained from the uncultured soil bacterium clone D30 (BGCs are drawn to approximate scale, color-coding is explained in the legend); (**b**) reconstructed biosynthetic pathways for 4-hydroxyphenylglycine (Hpg), 3,5-dihydroxyphenylglycine (Dpg), and β-hydroxytyrosine (Bht) encoded within *aba* (the Hpg biosynthesis pathway lacks 4-hydroxymandelate oxidase, as the corresponding gene is absent in *aba*); (**c**) putative structure of *A. bartoniae* GPA inferred from in silico analysis (the substrate and/or donor specificity of some tailoring enzymes remains unknown).

The borders of the antiSMASH-identified GPA-biosynthesis-related region were manually re-evaluated, resulting in the identification of a 46-gene (78,363 bp) BGC, further referred to as *aba* (Figure 1a, Table 1). One readily observable feature—three genes for sulfotransferases (as well as some other features, discussed below)—makes *aba* similar to the GPA BGC TEG (from the uncultured soil bacterium clone D30) [40] (Figure 1a). A detailed comparison of *aba* with known GPA BGCs and environmental GPA BGCs (previously employed as a source of tailoring enzymes) (27 BGCs, Table S2) enabled the assignment of functions in GPA biosynthesis to the majority of *aba* genes (discussed below).

**NRPS genes**. *abaA*, *abaB*, *abaC*, and *abaD* encode a typical 7-modular NRPS (Figure S2) [41]. The non-ribosomal codes of each adenylation (A) domain predict the following structure for the heptapeptide core: NH₂-AA1 (4-hydroxyphenylglycine, Hpg)-AA2 (β-hydroxytyrosine, Bht)-AA3 (3,5-dihydroxyphenylglycine, Dpg)-AA4 (Hpg)-AA5 (Hpg)-AA6 (Bht)-AA7 (Dpg)-COOH (Figure S2a). Thus, the heptapeptide produced by Aba NRPS is identical to the cores of many other GPAs from *Amycolatopsis* spp. (e.g., ristocetin [23,42] or GP1416 [24]).

Considering this, along with the organization of epimerization (E) domains in Aba NRPS (Figure S2b), it is reasonable to assume that the stereochemical configuration of *A. bartoniae* GPA would not differ from those described for other GPAs: NH_2_-d-d-l-d-d-l-l-COOH (e.g., [43]).

**Genes for aromatic amino acid biosynthesis enzymes.** Like all other known GPA BGCs, *aba* carries genes for the biosynthesis of Hpg, Dpg, and Bht [3], as well as several genes from the tyrosine biosynthetic pathway to enhance the supply of 4-hydroxyphenylpyruvate and tyrosine—crucial precursors for the biosynthesis of the non-proteinogenic amino acids Hpg, Dpg, and Bht [44] (Figure 1a,b). Aba30 (type III polyketide synthase), Aba31, Aba33 (enoyl-CoA-hydratases), and Aba32 (3,5-dihydroxyphenylacetyl-CoA 1,2-dioxygenase)—orthologues of the well-studied DpgA, DpgB, DpgD, and DpgC, respectively—are most likely involved in the production of 3,5-dihydroxyphenylglyoxylate [45,46] (Figure 1b). The latter is transformed into Dpg via the action of Aba25 (transaminase, HpgT orthologue) [46,47] (Figure 1b).

Next, Aba34, Aba5, and Aba6 are predicted as 3-deoxy-d-arabinoheptulosonate 7-phosphate (DAHP) synthase, chorismate mutase (CM), and prephenate dehydrogenase (PDH), respectively. Aba34 (DAHP synthase) catalyzes the initial step of tyrosine biosynthesis, converting d-erythrose 4-phosphate and 2-phosphoenolpyrvate into 3-deoxy-d-arabinoheptulosonate 7-phosphate, which is further converted by non-BGC-encoded enzymes into chorismate (Figure 1b). Aba34 belongs to an Iα subtype DAHP synthases (like other such enzymes from *Amycolatopsis*-derived GPA BGCs, Figure S3) [48]; Aba6 is related to *Amycolatopsis*-derived counterparts as well (Figure S4). Aba5 (CM) then transforms chorismate into prephenate, which is subsequently converted into 4-hydroxyphenylpyruvate by Aba6 (PDH) (Figure 1b). The latter enters the Hpg biosynthesis pathway, where it is first transformed into 4-hydroxymandelate by Aba26 (4-hydroxymandelate synthase, HmaS orthologue) (Figure 1b).

In all other GPA biosynthetic pathways, 4-hydroxymandelate is converted into 4-hydroxybenzoylformate by a 4-hydroxymandelate oxidase (Hmo orthologues) [49]. However, *aba* lacks the gene for 4-hydroxymandelate oxidase, nor could it be found outside the BGC (Figure 1a). Consequently, 4-hydroxybenzoylformate cannot be further transformed into Hpg by the action of Aba25 (transaminase).

Finally, *aba* encodes Aba35, Aba36, and Aba37, a thioesterase (Bhp orthologue), a single-modular NRPS (BpsD orthologue), and a monooxygenase (OxyD orthologue), respectively), which are necessary for the “off-line” production of Bht, later incorporated into the nascent heptapeptide [50,51,52].

**Cross-linking monooxygenase and halogenase genes.** Four monooxygenases, putatively involved in aglycone cross-linking, are encoded within *aba*. Phylogenetic reconstruction of these and other monooxygenases from GPA BGCs (Figure S5) suggests the following roles: Aba9 most likely catalyzes the formation of the AA2-AA4 cross-link (*D*-*O*-*E*, OxyA orthologue [53]); Aba10 catalyzes the AA1-AA3 cross-link (*F*-*O*-*G*, OxyE orthologue [54]); Aba11 catalyzes the AA4-AA6 cross-link (*C*-*O*-*D*, OxyB orthologue [53]); and Aba15 catalyzes the AA5-AA7 cross-link (*A-B*, OxyC orthologue [53]) (Figure 1c).

A single halogenase gene—*aba16*—was found in *aba*. To predict putative substrate specificity of Aba16, we have reconstructed its phylogeny together with other GPA BGC-encoded halogenases. We found out, that Aba16 forms a single well-separated clade with RSN28345 from *Amycolatopsis* sp. WAC 01416, the producer of GP1416 [24] (Figure S6). Thus, Aba16 presumably acts on AA2 and AA6 Bht residues (Figure 1c).

As *aba* does not encode any genes for the attachment of an aliphatic side chain, it is concluded that *A. bartoniae* GPA belongs to structural type III, possessing aglycone structure and halogenation pattern identical to GP1416.

**Methyltransferase genes**. *aba* carries a single gene for a class I S-adenosylmethionine (SAM)-dependent methyltransferase: Aba20. Phylogenetic reconstruction (Figure S7) places Aba20 in a clade with Orf23 (KFZ77414) from ristocetin BGC (*Amycolatopsis* sp. MJM2582 [23]), which has been experimentally shown to C-methylate AA3 (Hpg) of the heptapeptide [55]. Therefore, it is reasonable to assume that the GPA produced by *A. bartoniae* will have the same site methylated.

**Sulfotransferase genes.** Three genes were identified in *aba* encoding sulfotransfer_1 superfamily (cl21551) enzymes: *aba13, aba14,* and *aba15*. As mentioned above, this is reminiscent of the TEG BGC (Figure 1a,c). While the substrate specificity of TEG sulfotransferases is known—they act on AA3 (Teg12), AA4 (Teg14), and AA6 (Teg13) of the teicoplanin aglycone [40]—phylogenetic reconstruction of 11 sulfotransferases from GPA BGCs (Figure S8) did not provide sufficient evidence to speculate on the substrate specificity of Aba13, Aba14, and Aba15.

Moreover, *aba* encodes another enzyme that might be involved in the sulfation of the GPA aglycone: Aba19, an arylsulfotransferase (ASST) superfamily (cl26042) enzyme. Interestingly, Aba19 has an orthologue encoded in the TEG BGC, Teg19, which was not identified as a sulfotransferase at the time of its discovery. ASST enzymes are extremely rare in the biosynthesis of antibiotics. However, Aba19 shares 39% amino acid sequence identity (aa s. i.) with the sulfotransferases LpmB and Cpz4, encoded in the BGCs for liposidomycins and caprazamycins [56,57], which are responsible for synthesizing the 5-amino-5-deoxyribose-2-sulfate moiety. More recent studies have demonstrated that various bacterial ASST enzymes are capable of sulfating polyphenols of plant origin [58].

**Glycosyltransferase genes.** All glycosyltransferases (GTFs) identified to date in GPA BGCs belong to either family 1 (GT1) or family 39 (GT39) [59]. Similarly, *aba* encodes one GT1-GTF (Aba23), two GT39-GTFs (Aba18 and Aba24), and notably, a GT2-GTF (Aba17).

Closer inspection of the amino acid sequences of the encoded GTFs revealed that only one, Aba24, is homologous to known GTFs from GPA BGCs. Aba24 is a mannosyltransferase most likely responsible for attaching a d-mannose residue to AA7 of the heptapeptide aglycone (Figure S9). The second GT39-GTF, Aba18, is distantly related to known GPA GT39-GTFs (Figure S9) but shares 50% aa s. i. with Orf19 (AAM77988) from the neocarzinostatin BGC (*ncs*) [60]. Neocarzinostatin is decorated with a deoxy aminosugar derived from d-mannose, although this residue is believed to be attached by NcsC6 (another *ncs*-encoded GTF) [60]. Interestingly, NcsC6 is a homolog of the GT2-GTF Aba17 (41% aa s. i.), which lacks homologs in other GPA BGCs or in antibiotic BGCs in general, except for NcsC6. Thus, d-mannose may serve as a donor for both Aba17 and Aba18, although the substrate specificity of these enzymes could not be predicted (Figure 1c).

The GT1-GTF Aba23 does not have homologs encoded in GPA BGCs either (Figure S10). However, a BLAST search against the non-redundant protein sequence database using Aba23 as a query, identified a set of nearly identical proteins encoded within the genomes of *Streptomyces* sp. NPDC053474, *Streptomyces* sp. YIM 121038, *Streptomyces* sp. XD-27, *Streptomyces* sp. AN091965, *Streptomyces achromogenes* NPDC014880, and *Amycolatopsis samaneae* CGMCC 4.7643 (Figure S10). Further analyses revealed that the corresponding genes are parts of previously unknown GPA BGCs (see below). Although it is not possible to predict the donor or the substrate specificity of Aba23, the presence of similar proteins encoded in multiple GPA BGCs suggests that it may have functional significance, potentially modifying the GPA aglycone in an as-yet-unknown way.

**Regulatory, resistance, and transporter genes**. *aba* carries two cluster-situated genes encoding StrR-like pathway-specific transcriptional regulators: *aba1* and *aba4*. Phylogenetic reconstruction demonstrated that only one of these regulators, Aba4, is orthologous to the other StrR-like regulators from GPA BGCs, while Aba1 appears more distantly related (Figure S11). As StrR-like regulators are present in every GPA BGC [61] and are key regulators of biosynthesis, Aba4 is likely the main transcriptional activator of GPA biosynthesis in *A. bartoniae*, although an accessory role for Aba1 cannot be excluded.

As previously shown [22], *aba* encodes orthologues of VanY, VanHAX, and VanRS: Aba2, Aba38-40, and Aba41-42, respectively. The complete genome assembly confirmed the unusual location of the *van* genes, which are placed at the opposite ends of the BGC (Figure 1a), in contrast to their arrangement in other GPA BGCs from *Amycolatopsis* spp. [22].

Finally, like other *Amycolatopsis*-derived GPA BGCs (e.g., [62]), *aba* includes a gene encoding an MdlB (MsbA)-like ABC transporter (*aba7*), located upstream of the NRPS genes. It is most likely that Aba7 functions as the primary GPA exporter in *A. bartoniae* (Figure S12).

**Genes of unknown function.** The *A. bartoniae* GPA BGC also contains several genes of unknown function. First, *aba21* encodes a homolog (78% aa s. i.) of the Teg18 protein coded in the TEG BGC [40]. Next, *aba27* and *aba29* encode pyrroloquinoline quinone-dependent glucose dehydrogenases. A homolog of Aba27/Aba29 (64% aa s. i.) is found in the kendomycin BGC from *Verrucosispora* sp. SCSIO 07399; however, its function in that context also remains unknown, as the knockout of the corresponding gene did not affect kendomycin production [63].

### 3.2. Novel aba-Encoded Glycosyltransferase Leads to a Set of Unusual GPA BGCs

**Figure 2:**
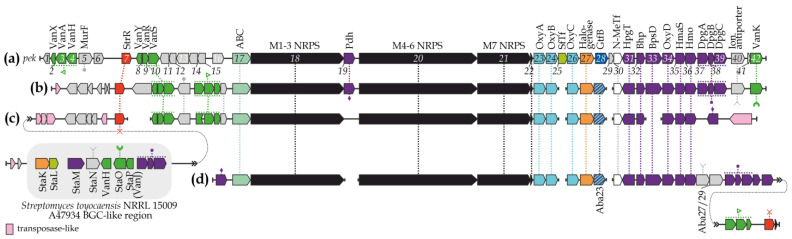
Genetic organization of the GPA BGCs from *Streptomyces* sp. WAC1420 (*pek*) (**a**), NPDC053474, YIM 121038, XD-27, and AN091965 (represented with a consensus scheme as their organization is identical (**b**), NPDC014880 (**c**), and *A. samaneae* CGMCC 4.7643 (**d**). Genes encoding homologous products are connected with dotted lines (SfTf—sulfotransferase; N-MeTf—N-methyltransferase). BGCs are drawn to approximate scale, for the color coding please refer to the legend of Figure 1.

An Aba23-mediated search identified previously unknown GPA BGCs in the genomes of *Streptomyces* sp. NPDC053474, *Streptomyces* sp. YIM 121038, *Streptomyces* sp. XD-27, *Streptomyces* sp. AN091965, *S. achromogenes* NPDC014880, and *A. samaneae* CGMCC 4.7643 (Table S2). In terms of genetic organization, the BGCs from NPDC053474, YIM 121038, XD-27 (despite containing several gaps due to low sequencing quality), and AN091965 were found to be identical (Figure 2b). However, the BGCs from *S. achromogenes* NPDC014880 and *A. samaneae* CGMCC 4.7643 differed both from these clusters and from each other (Figure 2c,d).

The organization of the NPDC053474, YIM 121038, XD-27, and AN091965 BGCs resembled that of the pekiskomycin BGC (*pek*) from *Streptomyces* sp. WAC1420 [64] (Figure 2a,b). In contrast, the NPDC014880 BGC was extensively rearranged and enriched with transposase-like genes, giving it a unique structure (Figure 2c).

The NRPSs encoded in this novel set of GPA BGCs exhibited a typical 7-modular organization (Figure S13, Table S3). The A-domains of Modules (M) 2 and 4–7 in these NRPSs were predicted to be specific for tyrosine, Hpg, Hpg, Bht, and Dpg, respectively (Figure S13, Table S3). However, the A-domain of M1 had an unknown non-ribosomal code (DAFYQGLVWK), which is most likely specific to an aliphatic amino acid (Figure S13, Table S3). Interestingly, the A-domain of M3 in NRPSs from NPDC053474, YIM 121038, XD-27, AN091965, and CGMCC 4.7643 shared the same non-ribosomal code (DVLLVGTIAK), identical to the non-ribosomal code of the pekiskomycin biosynthesis NRPS M3, which incorporates a glutamic acid residue [64]. By contrast, the A-domain of M3 in the NRPS from NPDC014880 had a distinct and unknown non-ribosomal code (DVQLMGSIAK). None of the identified BGCs encoded an OxyE orthologue (Figure 2, Table S3), although genes for OxyA, OxyB, and OxyC orthologues were present in each BGC (Figure S5).

The BGCs from NPDC053474, YIM 121038, XD-27, AN091965, and CGMCC 4.7643 each carried a single halogenase gene, while two such genes were identified in the NPDC014880 BGC (Table S3). Phylogenetic reconstruction revealed that the halogenases from NPDC053474, YIM 121038, XD-27, and AN091965 formed a single clade with the halogenases from the *pek* BGC (Figure S6). In contrast, the halogenase from the CGMCC 4.7643 BGC and one of the halogenases from the NPDC014880 BGC were related to the halogenase encoded in the avoparcin BGC from *Amycolatopsis coloradensis* DSM 44225 (Figure S6). Finally, the second halogenase encoded in the NPDC014880 BGC formed a distinct clade with StaK, an inactive halogenase from the *Streptomyces toyocaensis* NRRL 15009 A47934 BGC (Figure S6) [65].

All BGCs (except for NPDC014880, see below) contained complete sets of genes for Hpg and Dpg production (with the exception of DpgD) as well as “off-line” Bht production, in addition to a gene for prephenate dehydrogenase (PDH) (Table S3). Additionally, the CGMCC 4.7643 BGC also included a gene for DAHP synthase (Table S3). An unusual situation was observed in the NPDC014880 BGC, which contained only two genes for “off-line” Bht production (Bhp and OxyD) (Table S3). However, a gene encoding a StaM orthologue (a β-hydroxylase, the single enzyme required for “on-line” Bht production [66]) was identified in this BGC (Table S3).

It is reasonable to assume that these novel GPA BGCs may encode the production of chlorinated Type I GPAs with unprecedented aglycone structures. No GTF genes, apart from *aba23* orthologues, were identified in this set of GPA BGCs. However, each BGC carried a gene encoding an N-methyltransferase (Table S3). A truncated gene for a StaL homolog (a sulfotransferase from the A47934 BGC) was also found in the NPDC014880 BGC (Table S3).

Genes encoding StrR-like pathway-specific regulators and GPA-exporters were also identified in this set of GPA BGCs (Table S3, Figures S11 and S12).

Phylogenetic reconstructions performed in this work (e.g., Figures S4–S7, S11, and S12) revealed that the majority of GPA biosynthetic enzymes and other proteins encoded in the NPDC053474, YIM 121038, XD-27, AN091965, NPDC014880, and CGMCC 4.7643 BGCs cluster with their counterparts from the pekiskomycin BGCs, and more broadly *Amycolatopsis*-derived BGCs. However, several proteins encoded in the NPDC014880 BGC stand out as exceptions. These include StaM (Figure S14), StaK (Figure S6), and a truncated StaL orthologues (see above). Closer inspection revealed that the corresponding genes encoding StaM, StaK, and the truncated StaL orthologues are co-localized at the 5′ end of the NPDC014880 BGC, along with genes encoding StaN (ion antiporter, Figure S15), StaO (VanK, accessory GPA resistance protein), StaP (VanJ, accessory GPA resistance protein) orthologues, and the *dpgA-B-C* operon. Products of the *dpgA-B-C* operon were also found to be most closely related to their counterparts from the A47934 BGC (Figure S16 and Figure 2c). This suggests that the NPDC014880 BGC may represent a recombinant BGC, combining elements of A47934-like and *pek*-like BGCs (Figure 2c). The acquisition of *pek*-like elements may have occurred through a horizontal gene transfer (HGT) event, supported by the presence of numerous transposase-related genes co-localized with the NPDC014880 BGC (Figure 2c).

### 3.3. aba-Encoded GPA Biosynthetic Pathway Is Inactive Under a Broad Range of Laboratory Conditions

The original paper reporting the isolation and description of *A. bartoniae* DSM 45807 did not include any information about its optimal cultivation conditions [67]. To address this, we first cultivated *A. bartoniae* DSM 45807 on a set of agar media (ISP1-7) and found that ISP5 supported optimal growth and abundant sporulation (Figure S17). Spore suspensions obtained from ISP5 cultures were used to inoculate (approximately 10⁷ spores per plate) 16 different agar media, including MM, TM1, SDA, SEA, MYM, PDA, YMPG, CzA, R5, and ISP1-7 (see Materials and Methods section). After 120 h of incubation, agar plugs were cut from the surfaces of the lawns to test for growth inhibition activity against *B. subtilis* HB0950, *E. coli* DH5α, and *D. hansenii* VKM Y-9. No growth inhibition was observed against *E. coli* DH5α or yeast (Figures S18a,b), but small growth inhibition halos were observed around agar plugs from SDA and R5 cultures against *B. subtilis* HB0950 (Figure S18c). However, the lack of X-Gal chromogenic conversion at the edges of these halos indicated that the observed activity was not GPA-related.

Next, we tested the antimicrobial properties of *A. bartoniae* DSM 45807 in various liquid media. The first step was identifying an optimal medium for vegetative culture by testing the following liquid media: SEED (used for vegetative culture of teicoplanin-producing *Act. teichomyceticus* KCCM 10601 [68]); TSB (used for vegetative culture of ristocetin-producing *A. japonica* MG417-CF17 [42]); E25 (used for vegetative culture of teicoplanin-producing *Act. teichomyceticus* ATCC 31121 [69]); E26 (used for vegetative culture of A40926-producing *N. gerenzanensis* ATCC 39727 [70]); and several basic actinobacterial cultivation media (YMPG, VSP, GYM). We found that the SEED medium supported the highest biomass accumulation for *A. bartoniae* DSM 45807 after 72 h of cultivation (approximately 100 g/L of fresh biomass and 10 g/L of dry biomass, Figure S19). No GPA-related activity was observed in *A. bartoniae* DSM 45807 cultures grown in the aforementioned media at 24, 48, or 72 h, as determined by the *B. subtilis* HB0950 growth inhibition assay (Figure S20a).

Finally, we tested *A. bartoniae* DSM 45807 under conditions previously shown to induce GPA production. To this aim, 72-h precultures grown in SEED medium were used to inoculate main cultures in R5 (used for ristocetin production in *A. japonica* MG417-CF17 [42]), TM1 (used for teicoplanin production in *Act. teichomyceticus* ATCC 31121 [69]), FM2 (used for A40926 production in *N. gerenzanensis* ATCC 39727 [70]), and ISP2 (used for A50926 production in *N. coxensis* DSM 45129 [71]). These cultures were grown for up to 168 h, with broth samples collected at regular 24-h intervals and tested in the *B. subtilis* HB0950 growth inhibition assay. No GPA-related activity was detected in any of the media at any tested time point (Figure S20b).

## 4. Discussion

Since the discoveries made during the pre-genomic and genomic eras, the arsenal of Type I-IV GPA tailoring enzymes has remained largely constant [55,72]. Consequently, the presence of several unprecedented putative tailoring enzymes, coded in *aba*, is quite surprising. These include novel GT1- and GT39-GTFs, a GT2-GTF, and an ASST-superfamily sulfotransferase. At the time of the initial discovery of the GPA BGC in *A. bartoniae* DSM 45807 [22], we doubted whether such a GPA BGC configuration was possible. However, the complete genome assembly of *A. bartoniae* DSM 45807 presented in this study has ruled out any sequencing artifacts, confirming the exotic organization of *aba*.

Pathway prediction suggests that the *A. bartoniae* GPA is a highly sulfated, halogenated, and methylated Type III compound (Figure 1c). While only one glycosylation site could be predicted with certainty—a d-mannose residue likely attached to AA7 of the aglycone, three additional GTFs encoded in *aba* may further extend the glycosylation pattern of the *A. bartoniae* GPA in an as-yet-unknown manner. Unfortunately, as indicated by our cultivation experiments, *aba*-encoded biosynthetic pathway appears to be inactive under a broad range of laboratory conditions, halting further investigation of GPA production in the native host. In silico analysis suggests a plausible cause for the lack of GPA production: *aba* lacks a gene for the 4-hydroxymandelate oxidase (Hmo), which is necessary for the production of Hpg, a key precursor for the NRPS assembly line. The genome of *A. bartoniae* DSM 45807 does encode four additional genes for α-hydroxy-acid oxidizing enzymes (34–45% aa s. i. with the Hmo encoded in the *Amycolatopsis balhimycina* DSM 5908 balhimycin BGC), but these genes do not appear capable of complementing the absence of the Hmo gene. Efforts to activate GPA production in *A. bartoniae* DSM 45807 may include additive feeding with 4-hydroxybenzoylformate or the heterologous expression of an Hmo gene. However, all our attempts to transfer plasmid DNA into *A. bartoniae* DSM 45807 cells have been unsuccessful so far. Despite these challenges, work to activate the *aba* BGC is ongoing in our laboratories.

Another outcome of this study was the discovery of five novel *Streptomyces*-derived GPA BGCs and a novel BGC from *A. samaneae* CGMCC 4.7643. All these BGCs carry *aba23*-like novel GT1-GTF genes and are likely to encode the biosynthesis of novel Type I methylated and halogenated GPAs. Additionally, these GPAs may be glycosylated if *aba23* functions as an active tailoring enzyme. To date, Type I-IV GPA BGCs from *Streptomyces* spp. were limited to the pekiskomycin BGCs from *Streptomyces* sp. WAC4229 and WAC1420 [64] and the A47934 BGC from *S. toyocaensis* NRRL 15009 [73]. The genus itself, however, has been found as an abundant source of Type V GPAs [20]. Our results suggest that *Streptomyces* spp. are likely a richer source of Type I GPAs than previously anticipated. Peculiarly, the GPA BGC from *S. achromogenes* NPDC014880 is an evident hybrid of *pek*-like and A47934-like BGCs. The genomic surroundings of this BGC are enriched with transposase-related genes, which are also present, albeit to a lesser extent, close to the other four new GPA BGCs from *Streptomyces* spp. This suggests that these BGCs may have participated in HGT events. Further investigation of the GPA BGC from *S. achromogenes* NPDC014880—and similar hybrid BGCs, if discovered—could provide new insights into the modular evolution of GPA BGCs. Moreover, if it is assumed that some GPA BGCs were delivered to *Streptomyces* spp. via an HGT event, it is peculiar how they integrate into the AdpA-centered global regulation of antibiotic production [74]. Overall, the global regulation of GPA production remains under-investigated [75], while *Streptomyces*-derived GPA BGCs could serve as a good model for studying this type of regulation, at least within *Streptomyces* spp.

To conclude, this study described novel GPA BGC configurations in *A. bartoniae* DSM 45807 and in several other new GPA BGCs, expanding the known diversity of GPAs BGCs. The exotic *aba* BGC, encoding several unique tailoring enzymes, presents an intriguing yet challenging target for activation and merits further investigation.

## Figures and Tables

**Table 1:** Genes identified within *aba* BGC with putative assigned functions in GPA biosynthesis.

aba	Locus Tag:	Product:	Function:
*aba1*	*AMYBAR_003213*	StrR-like transcriptional regulator	Function unknown
*aba2*	*AMYBAR_003214*	M15 family metallopeptidase (VanY)	Cleaves d-Ala-d-Ala dipeptides, involved in GPA resistance
*aba3*	*AMYBAR_003215*	KefB membrane component of Kef-type K^+^ transport system	Putatively involved in inorganic ion transport
*aba4*	*AMYBAR_003216*	StrR-like transcriptional regulator	Putatively involved in the positive regulation of GPA biosynthesis
*aba5*	*AMYBAR_003217*	Chorismate mutase type I	Converts chorismate to prephenate, involved in amino acid supply for GPA biosynthesis
*aba6*	*AMYBAR_003218*	Prephenate dehydrogenase (PDH)	Converts prephenate to 4-hydroxyphenylpyruvate, involved in amino acid supply for GPA biosynthesis
*aba7*	*AMYBAR_003219*	MdlB(MsbA)-like ABC-transporter	Putatively involved in GPA export
*abaA*	*AMYBAR_003220*	NRPS	GPA biosynthesis NRPS, modules 1–2
*abaB*	*AMYBAR_003221*	NRPS	GPA biosynthesis NRPS, module 3
*abaC*	*AMYBAR_003222*	NRPS	GPA biosynthesis NRPS, modules 4–5–6
*abaD*	*AMYBAR_003223*	NRPS	GPA biosynthesis NRPS, module 7
*aba8*	*AMYBAR_003224*	MbtH-like protein	GPA biosynthesis NRPS chaperone
*aba9*	*AMYBAR_003225*	Cytochrome P450	GPA cross-linking monooxygenase (OxyA), involved in the formation of *D*-*O*-*E* link
*aba10*	*AMYBAR_003226*	Cytochrome P450	GPA cross-linking monooxygenase (OxyE), involved in the formation of *F*-*O*-*G* link
*aba11*	*AMYBAR_003227*	Cytochrome P450	GPA cross-linking monooxygenase (OxyB), involved in the formation of *C*-*O*-*D* link
*aba12*	*AMYBAR_003228*	Sulfotransferase_1 superfamily domain protein	Putatively involved in GPA sulfation
*aba13*	*AMYBAR_003229*	Sulfotransferase_1 superfamily domain protein	Putatively involved in GPA sulfation
*aba14*	*AMYBAR_003230*	Sulfotransferase_1 superfamily domain protein	Putatively involved in GPA sulfation
*aba15*	*AMYBAR_003231*	Cytochrome P450	GPA cross-linking monooxygenase (OxyC), involved in the formation of *A*-*B* link
*aba16*	*AMYBAR_003232*	Halogenase	Involved in GPA halogenation
*aba17*	*AMYBAR_003233*	GT2-family glycosyltransferase	Function unknown
*aba18*	*AMYBAR_003234*	GT39-family glycosyltransferase	Function unknown
*aba19*	*AMYBAR_003235*	Arylsulfotransferase (ASST)	Function unknown
*aba20*	*AMYBAR_003236*	Methyltransferase	Involved in C-methylation of the aglycone AA3
*aba21*	*AMYBAR_003237*	Hypothetical protein	Function unknown
*aba22*	*AMYBAR_003238*	Dyp-type peroxidase family protein	Function unknown
*aba23*	*AMYBAR_003239*	GT1-family glycosyltransferase	Function unknown
*aba24*	*AMYBAR_003240*	GT39-family glycosyltransferase	Attaches d-mannose residue to GPA aglycone AA7
*aba25*	*AMYBAR_003241*	4-hydroxyphenylglycine transaminase (HpgT)	Converts 4-hydroxybenzoylformate to 4-hydroxyphenylglycine or 3,5-dihydroxyphenylglyoxylate to 3,5-dihydroxyphenylglycine, involved in the biosynthesis of Hpg and Dpg
*aba26*	*AMYBAR_003242*	Hydroxymandelate synthase (HmaS)	Converts 4-hydroxyphenylpyruvate to 4-hydroxymandelate, involved in Hpg biosynthesis
*aba27*	*AMYBAR_003243*	Pyrroloquinoline quinone-dependent glucose dehydrogenase	Function unknown
*aba28*	*AMYBAR_003244*	KefB membrane component of Kef-type K^+^ transport system	Putatively involved in inorganic ion transport
*aba29*	*AMYBAR_003245*	Pyrroloquinoline quinone-dependent glucose dehydrogenase	Function unknown
*aba30*	*AMYBAR_003246*	Type III polyketide synthase (DpgA)	Converts malonyl-CoA units into 3,5-dihydroxyphenylacetate, involved in Dpg biosynthesis
*aba31*	*AMYBAR_003247*	Enoyl-CoA hydratase (DpgB)	Converts malonyl-CoA units into 3,5-dihydroxyphenylacetate, involved in Dpg biosynthesis
*aba32*	*AMYBAR_003248*	3,5-dihydroxyphenylacetyl-CoA 1,2-dioxygenase (DpgC)	Converts 3,5-dihydroxyphenylacetate in 3,5-dihydroxyphenylglyoxylate, involved in Dpg biosynthesis
*aba33*	*AMYBAR_003249*	Enoyl-CoA hydratase (DpgD)	Converts malonyl-CoA units into 3,5-dihydroxyphenylacetate, involved in Dpg biosynthesis
*aba34*	*AMYBAR_003250*	3-deoxy-d-arabinoheptulosonate 7-phosphate synthase (Dahp)	Converts phosphoenolpyruvate and d-erythrose-4-phosphate into 3-deoxy-d-arabinoheptulosonate 7-phosphate, involved in amino acid supply for GPA biosynthesis
*aba35*	*AMYBAR_003251*	Thioesterase	Catalyzes Bht release from NRPS, involved in Bht biosynthesis
*aba36*	*AMYBAR_003252*	Single-modular NRPS (Bht biosynthesis)	Carries tyrosine and Bht, involved in Bht biosynthesis
*aba37*	*AMYBAR_003253*	Cytochrome P450	Converts tyrosine to Bht, involved in Bht biosynthesis
*aba38*	*AMYBAR_003254*	d-Lactate dehydrogenase (VanH)	Converts pyruvate to d-lactate, involved in GPA resistance
*aba39*	*AMYBAR_003255*	d-Ala-d-Lac ligase (VanA)	Produces d-Ala-d-Lac depsipeptide, involved in GPA resistance
*aba40*	*AMYBAR_003256*	d,d-dipeptidase (VanX)	Cleaves d-Ala-d-Ala dipeptides, involved in GPA resistance
*aba41*	*AMYBAR_003257*	Two-component regulatory system sensor histidine kinase (VanS)	Involved in GPA sensing and resistance
*aba42*	*AMYBAR_003258*	Two-component regulatory system sensor resistance response regulator (VanR)	Involved in GPA sensing and resistance

## Data Availability

Complete genome sequence of *A. bartoniae* DSM 45807 was submitted to GenBank under the accession number CP174150.

## Supplementary Materials

The following supporting information can be downloaded at: https://www.mdpi.com/article/10.3390/genes15121651/s1, including: Compositions of cultivation media used in the work. Figure S1: Microheterogeneity of *A. bartoniae* DSM 45807 16S rRNA genes. Figure S2: Organization of *A. bartoniae* DSM 45807 NRPS. Figure S3: Phylogeny of 23 DAHP synthases coded in various GPA BGCs. Figure S4: Phylogeny of 34 PDHs coded in various GPA BGCs. Figure S5: Phylogeny of 119 cross-linking monooxygenases coded in various GPA BGCs. Figure S6: Phylogeny of halogenases coded in various GPA BGCs. Figure S7: Phylogeny of 35 methyltransferases coded in various GPA BGCs. Figure S8: Phylogeny of 11 sulfotransferases coded in various GPA BGCs. Figure S9: Phylogeny of GT1-GTFs coded in various GPA BGCs. Figure S10: Phylogeny of 23 GT39-GTFs coded in various GPA BGCs. Figure S11: Phylogeny of StrR-like transcriptional regulators coded in various GPA BGCs. Figure S12: Phylogeny of ABC-transporters coded in various GPA BGCs. Figure S13: Organization of NRPSs coded in a set of 6 newly discovered GPA BGCs. Figure S14: Phylogeny of 9 β-hydroxylases coded in various GPA BGCs. Figure S15: Phylogeny of 29 membrane ion antiporters s coded in various GPA BGCs. Figure S16: Phylogeny of DpgA, DpgB, and DpgC proteins coded in various GPA BGCs. Figure S17: *A. bartoniae* DSM 45807 sporulation. Figure S18: *Escherichia coli* DH5α, *Debaryomyces hansenii* VKM Y-9, and *Bacillus subtilis* HB0950 growth inhibition assays using agar plugs taken from *A. bartoniae* DSM 45807 lawns. Figure S19: *A. bartoniae* DSM 45807 biomass accumulation in various liquid media. Figure S20: *A. bartoniae* DSM 45807 does not exhibit GPA-related activities when cultivated in submerged culture. Table S1: antiSMASH analysis of *A. bartoniae* DSM 45807 genome. Table S2: Summary of 33 GPA BGCs used in the analysis. Table S3: List of key proteins encoded in a set of 6 newly discovered GPA BGCs. Supplementary references: [76,77,78,79,80,81].
