# Supplementary material for: Complete Genome Assembly of Amycolatopsis bartoniae DSM 45807T Allows the Characterization of a Novel Glycopeptide Biosynthetic Gene Cluster
Source: Genes (Basel). 2024 Dec 22;15(12):1651. doi: 10.3390/genes15121651 (PMC11727664; doi:10.3390/genes15121651)
Supplement: Supplementary file 1 [file genes-15-01651-s001.zip › genes-3377810-supplementary.pdf]

Electronic Supplementary Material

Complete genome assembly of *Amycolatopsis bartoniae* DSM 45807[T] allows the characterization of a novel glycopeptide biosynthetic gene cluster

Anastasia Stepanyshyn [1], Christian Rückert-Reed [2,3], Tobias Busche [2], Bohdan Yaruta [1], Andres Andreo-Vidal [4], Flavia Marinelli [4], Jörn Kalinowski [2], and Oleksandr Yushchuk [1*]

[1] Department of Genetics and Biotechnology, Ivan Franko National University of Lviv, 79005 Lviv, Ukraine; nastia.stepanyshyn@gmail.com (A.S.); 3bogdanyaryta3@gmail.com (B.Y.); oleksandr.yushchuk@lnu.edu.ua (O.Y.);

[2] Technology Platform Genomics, CeBiTec, Bielefeld University, Sequenz 1, 33615 Bielefeld, Germany; chris-tian.rueckert@uni-bielefeld.de (C.R-R.); tobias.busche@uni-bielefeld.de (T.B.); joern@cebitec.uni-bielefeld.de (J.K.);

[3] Medical School OWL, Bielefeld University, Sequenz 1, 33615 Bielefeld, Germany;

[4] Department of Biotechnology and Life Sciences, University of Insubria, 21100 Varese, Italy; andresandre-oiv@gmail.com (A.A.-V.); flavia.marinelli@uninsubria.it (F.M.);

* Correspondence: oleksandr.yushchuk@lnu.edu.ua; Tel.: +38 (032) 239 47 68.

Inventory of Supplementary Items

Supplementary Item:	Page:
**Compositions of cultivation media used in the work**	3
**Supplementary tables**	4
**Table S1.** antiSMASH analysis of *Amycolatopsis bartoniae* DSM 45807 genome.	4
**Table S2.** Summary of 33 GPA BGCs used in the analysis.	6
**Table S3.** List of key proteins encoded in a set of 6 newly discovered GPA BGCs.	7
**Supplementary figures**	9
**Figure S1.** Microheterogeneity of *A. bartoniae* DSM 45807 16S rRNA genes.	9
**Figure S2.** Organization of *A. bartoniae* DSM 45807 NRPS.	9
**Figure S3.** Phylogeny of 23 DAHP synthases coded in various GPA BGCs.	10
**Figure S4.** Phylogeny of 34 PDHs coded in various GPA BGCs.	11
**Figure S5.** Phylogeny of 119 cross-linking monooxygenases coded in various GPA BGCs.	12
**Figure S6.** Phylogeny of halogenases coded in various GPA BGCs.	13
**Figure S7.** Phylogeny of 35 methyltransferases coded in various GPA BGCs.	14
**Figure S8.** Phylogeny of 11 sulfotransferases coded in various GPA BGCs.	15
**Figure S9.** Phylogeny of 23 GT39-GTFs coded in various GPA BGCs.	16
**Figure S10.** Phylogeny of GT1-GTFs coded in various GPA BGCs.	17
**Figure S11.** Phylogeny of StrR-like transcriptional regulators coded in various GPA BGCs.	18
**Figure S12.** Phylogeny of ABC-transporters coded in various GPA BGCs.	19
**Figure S13.** Organization of NRPSs coded in a set of 6 newly discovered GPA BGCs.	20
**Figure S14.** Phylogeny of 9 β-hydroxylases coded in various GPA BGCs.	20
**Figure S15.** Phylogeny of 29 membrane ion antiporters s coded in various GPA BGCs.	21
**Figure S16.** Phylogeny of DpgA, DpgB, and DpgC proteins coded in various GPA BGCs.	22
**Figure S17.** *A. bartoniae* DSM 45807 sporulation.	23
**Figure S18.** *Escherichia coli* DH5α, *Debaryomyces hansenii* VKM Y-9, and *Bacillus subtilis* HB0950 growth inhibition assays using agar plugs taken from *A. bartoniae* DSM 45807 lawns.	23
**Figure S19.** *A. bartoniae* DSM 45807 biomass accumulation in various liquid media.	24
**Figure S20.** *A. bartoniae* DSM 45807 doesn't exhibit GPA-related activities when cultivated in submerged culture.	25
**Supplementary References**	26

Compositions of cultivation media used in the work

MM [1], g/L of distilled water: L-Asparagine – 0.5; K_2HPO_4 – 0.5; $MgSO_4 \times 7H_2O$ – 0.2; $FeSO_4 \times 7H_2O$ – 0.01; Glucose – 10; Agar – 10; pH 7.0-7.2.

Soil extract agar (**SEA**, DSMZ Medium 12, https://www.dsmz.de/microorganisms/medium/pdf/DSMZ_Medium12.pdf), g/L of tap water: Air dried garden soil – 400; Agar - 15g; pH 6.8-7.0.

MYM [3], g/L of distilled water: Yeast extract – 4; Maltose – 4; Malt extract – 10; Agar – 20; pH 7.5.

YMPG [3], g/L of distilled water (prepared without agar for submerged cultivation): Yeast extract – 4; Bacto peptone – 1; Malt extract – 10; Glucose – 10; $MgCl_2 \times 6H_2O$ – 2; Agar – 20; pH 7.0.

Czapek agar (**CzA**, NRRL Medium No. 8, https://nrrl.ncaur.usda.gov/media/84/download)/, g/L of distilled water: Soluble starch – 20; $NaNO_3$ – 1; K_2HPO_4 – 0.5; $MgSO_4$ – 0.5; KCl– 0.5; $FeSO_4$ – 0.001; Agar – 15; pH 7.0.

R5 [1], g/900 mL of deionized water (prepared without agar for submerged cultivation): Saccharose – 103; Glucose – 10; K_2SO_4 – 0.25; $MgCl_2 \times 6H_2O$ – 10.12; Casamino acids – 0.1; Yeast extract – 5; TES buffer – 5.73; Trace elements solution (mg/L: $ZnCl_2$ – 40; $FeCl_3 \times 6H_2O$ – 200; $CuCl_2 \times 2H_2O$ – 10; $MnCl_2 \times 4H_2O$ – 10; $Na_2B_4O_7 \times 10H_2O$ – 10; $(NH_4)_6Mo_7O_{24} \times 4H_2O$ – 10) – 2 mL; Agar – 20; pH 7.2. Added at the time of use: $CaCl_2$ 3.68% (w/v) – 80 mL; KH_2PO_4 0.54% (w/v) – 10 mL; L-Proline 20% (w/v) – 15 mL.

ISP1 [1], g/L of distilled water: Tryptone – 5.0; Yeast extract – 3.0; Agar – 15; pH 7.5.

ISP2 [1], g/L of distilled water (prepared without agar for submerged cultivation): Yeast extract – 4; Malt extract – 10; Dextrose – 4; Agar – 20; pH 7.5.

ISP3 [1], g/L of tap water: Fine ground whole oats (Kozub, Poltava, Ukraine) – 34; Agar – 20; pH 7.5.

ISP4 [1], g/L of distilled water: Soluble starch – 10; $CaCO_3$ – 2; K_2HPO_4 (anhydrous) – 1; $MgSO_4 \times 7H_2O$ – 1; NaCl – 1; $(NH_4)_2SO_4$ – 2; $FeSO_4 \times 7\ H_2O$ – 0.001; $MnCl_2 \times 4\ H_2O$ – 0.001; $ZnSO_4 \times 7\ H_2O$ – 0.001; Agar – 20; pH 7.0 – 7.4.

ISP5 [1], g/L of distilled water: L-Asparagine – 1; K_2HPO_4 – 1; glycerol – 10; $FeSO_4 \times 7H_2O$ – 0.001; $MnCl_2 \times 4H_2O$ – 0.001; $ZnSO_4 \times 7H_2O$ – 0.001; Agar – 20; pH 7.0.

ISP6 [1], g/L of distilled water: Soytone – 15; Peptone – 5; Ferric ammonium citrate – 0.5; K_2HPO_4 – 1; $Na_2S_2O_3$ – 0.08; Yeast extract – 1; Agar – 20; pH 7.5.

ISP7 [1], g/L of distilled water: L-Asparagine – 1; L-Tyrosine – 0.5; K_2HPO_4 – 0.5; $MgSO_4 \times 7H_2O$ – 0.5; NaCl – 0.5; Trace element solution (mg/mL, $FeSO_4 \times 7H_2O$ – 1.36; $CuCl_2 \times 2H_2O$ – 0.027; $CoCl_2 \times 6H_2O$ – 0.04; $Na_2MoO_4 \times 2H_2O$ – 0.025; ZnCl2 – 0.02; H_3BO_3 – 2.85; $MnCl_2 \times 4H_2O$ – 1.8; $Na_2C_4H_4O_6$ – 1.77) – 1 mL; Agar – 20; pH 7.3.

SEED [4], g/L of distilled water: Glucose – 30; Yeast extract – 5; Peptone – 5; KH_2PO_4 – 2; K_2HPO_4 – 4; $MgSO_4$ – 0.5; pH 7.5.

E25 [2], (g/L of distilled water): Dextrose – 25; Meat extract – 4; Yeast extract – 1; Soybean flour – 10; Bacto peptone – 4; NaCl – 2.5; pH 7.5.

E26 [5], (g/L of distilled water): Dextrose – 25; Soy flour – 20; Yeast extract – 4; NaCl – 1.25; $CaCO_3$ – 5; pH 7.5.

VSP [5], (g/L of distilled water): Soluble starch (Difco, Franklin Lakes, NJ, USA) – 24; Dextrose – 1; Meat extract – 3; Yeast extract – 5; Tryptose – 5; L-proline – 0.5; Saccharose – 50; pH 7.5.

GYM [6], g/L of distilled water (prepared without agar for submerged cultivation): Yeast extract – 4; Malt extract – 10; Dextrose – 4; $CaCO_3$ – 2; pH 7.5.

TM1 [2], g/L of distilled water (prepared without agar for submerged cultivation): Dextrose – 10; Malt extract – 30; Yeast extract – 2.5; Soybean flour – 15; $CaCO_3$ – 4; pH 7.5.

FM2 [5], (g/L of distilled water): Dextrose – 30; Soy flour – 30; Yeast extract – 8; Malt extract – 15; $CaCO_3$ – 5; L-valine – 1; pH 7.5.

Supplementary Tables

Table S1. Results of antiSMASH [7] analysis conducted on *Amycolatopsis bartoniae* DSM 45807 genome.

Location (bp):	Most similar BGC (% genes showing similarity), MIBiG accession (in case the similarity involves core biosynthetic genes):	Comments:
**(1)**	**(2)**	**(3)**
531,316 - 602,286	Siderophore amychelin A BGC from *Amycolatopsis methanolica* 239 (72%), BGC0002544	-
888,300 - 932,505	*n/i**	Parts of the region homologous to: pyridomycin BGC (BGC0001039) from *Streptomyces pyridomyceticus* NRRL B-2517; fluostatin M BGC (BGC0001596) from *Streptomyces albus* DSM 41398
2,271,081 - 2,293,225	Geosmin biosynthesis gene from *Streptomyces coelicolor* A3(2) (100%), BGC0001181	-
2,349,903 - 2,383,748	ε-Poly-L-lysine biosynthesis gene from *Epichloe festucae* (100%), BGC0002174	-
3,101,686 - 3,127,535	Isorenieratene BGC from *Streptomyces collinus* Tü 365 (36%), BGC0001227	-
3,239,844 - 3,312,533	Dechlorocuracomycin BGC from *Streptomyces noursei* ATCC 11455 (12%), BGC0001569	Genes for 9 modular NRPS
3,354,385 - 3,457,460	-	GPA BGC (*aba*)
3,753,805 - 3,774,860	Cattleyene biosynthetic gene cluster from *Streptomyces cattleya* NRRL 8057 (50%), BGC0002309	-
4,449,752 - 4,475,762	Hopene biosynthetic gene cluster from *Streptomyces coelicolor* A3(2) (46%), BGC0000663	-
5,006,430 - 5,028,483	Lankacidin C biosynthetic gene cluster from *Streptomyces rochei* plasmid pSLA2-L (26%), BGC0001100	No PKS or NRPS genes
5,033,193 - 5,067,551	*n/i*	Class III lanthionine synthetase genes
5,166,200 - 5,210,519	*n/i*	1 Module NRPS gene
5,210,784 - 5,272,962	Enteromycin biosynthetic gene cluster from *Streptomyces achromogenes* ssp. *streptozoticus* (29%), BGC0002499	Type III PKS gene
5,322,665 - 5,385,434	Luminmycin A biosynthetic gene cluster from *Photorhabdus laumondii* ssp. *laumondii* TTO1 (15%), BGC0000383	No PKS or NRPS genes
5,569,436 - 5,593,691	*n/i*	Class I lanthipeptide biosynthesis genes
5,596,516 - 5,639,494	Collismycin A biosynthetic gene cluster from *Streptomyces* sp. CS40 (18%), BGC0000973	1 Module PKS gene

(1)	(2)	(3)
5,749,430 - 5,779,178	Nonactin biosynthetic gene cluster from *Streptomyces griseus* DSM 40695 (33%), BGC0000244	-
5,988,890 - 6,010,357	*n/i*	Mycofactocin-like RiPP biosynthetic genes
7,284,662 - 7,295,054	Ectoine biosynthetic gene cluster from *Streptomyces anulatus* (100%), BGC0000853	-
7,494,032 - 7,554,743	Ery-9 biosynthetic gene cluster from *Saccharopolyspora erythraea* NRRL 2338 (100%), BGC0000513	-

**n/i* – not identified.

Table S2. Summary of 33 GPA BGCs, in addition to the *aba* BGC, used in this work for comparative *in silico* analysis.

	Organism	Accession number	Product
1	*Actinoplanes teichomyceticus* ATCC 31121	AJ632270	Teicoplanin
2	*Actinoplanes* sp. ATCC 53533	KF192710	UK-68,597
3	*Amycolatopsis balhimycina* DSM 5908	Y16952	Balhimycin
4	*Amycolatopsis coloradensis* DSM 44225	MQUQ01000006	Avoparcin
5	*Amycolatopsis decaplanina* DSM 44594	AOHO01000074	Decaplanin
6	*Amycolatopsis keratiniphila* HCCB10007	CP003410	Vancomycin
7	*Amycolatopsis keratiniphila* ssp. *keratiniphila* DSM 44409	MH428036	Keratinimicin
8	*Amycolatopsis keratiniphila* subsp. *nogabecina* FH 1893	LT629789	Nogabecin
9	*Amycolatopsis orientalis* B-37	CP016174	Norvancomycin
10	*Amycolatopsis japonica* MG417-CF17	CP008953	Ristocetin
11	*Amycolatopsis lurida* NRRL 2430	KJ364518	Ristocetin
12	*Amycolatopsis* sp. MJM2582	JPLW01000007	Ristocetin
13	*Amycolatopsis* sp. TNS106	CP024972	Ristocetin
14	*Amycolatopsis* sp. WAC 01416	QHHX01000008	GP1416
15	*Amycolatopsis* sp. WAC 04169	QHJI01000006	Dimethylvanco mycin
16	Uncultured organism CA37	HM486074	?
17	Uncultured organism CA878	HM486075	?
18	Uncultured organism CA915	HM486076	?
19	Uncultured organism esnapd15	KF264554	?
20	*Kibdelosporangium aridum* A82846	QHKI01000005	Chloroeremomy cin
21	*Nonomuraea gerenzanensis* ATCC 39727	AJ561198	A40926
22	*Nonomuraea coxensis* DSM 45129	CP068985	A50926
23	*Streptomyces toyocaensis* NRRL 15009	U82965	A47934
24	*Streptomyces* sp. WAC1420	JX026280	Pekiskomycin
25	*Streptomyces* sp. WAC4229	KC688274	Pekiskomycin
26	Uncultured soil bacterium clone D30 TEG gene cluster	EU874253	?
27	Uncultured soil bacterium clone B128 VEG gene cluster	EU874252	?
GPA BGCs identified in this work:			
28	*Streptomyces* sp. NPDC053474	JBITXV010000023	?
29	*Streptomyces* sp. YIM 121038	NZ_CP030771	?
30	*Streptomyces* sp. XD-27	NZ_CP130713	?
31	*Streptomyces* sp. AN091965	NZ_JALDMY010000001	?
32	*Amycolatopsis samaneae* CGMCC 4.7643	NZ_JBHUKU010000022	?
33	*Streptomyces achromogenes* NPDC014880	NZ_JBIBWN010000029	?

Table S3. List of key proteins encoded in a set of 6 newly discovered GPA BGCs from *Streptomyces* sp. NPDC053474, *Streptomyces* sp. YIM 121038, *Streptomyces* sp. XD-27, *Streptomyces* sp. AN091965, *S. achromogenes* NPDC014880, and *A. samaneae* CGMCC 4.7643, as well as counterparts from and *Streptomyces* sp. WAC1420 *pek* BGC and *S. toyocaensis* NRRL 15009 A47934 BGC.

Protein	*Streptomyces* sp. NPDC053474	*Streptomyces* sp. YIM 121038	*Streptomyces* sp. XD-27	*Streptomyces* sp. AN091965	*Streptomyces achromogenes* NPDC014880	*Amycolatopsis samaneae* CGMCC 4.7643	*Streptomyces* sp. WAC1420 *pek*	*Streptomyces toyocaensis* NRRL 15009 A47934 BGC
	**(1)**	**(2)**	**(3)**	**(4)**	**(5)**	**(6)**	**(7)**	**(8)**
	MFI8933340	WP_138963346	WP_304459974	WP_242714308	WP_391632540	WP_345386643	Pek18	
Module 1	DAFYQGLVWK (Leu, 68%)	DAFYQGLVWK (Leu, 68%)	DAFYQGLVWK (Leu, 68%)	DAFYQGLVWK (Leu, 68%)	DAFYQGLVWK (Leu, 65%)	DAFYQGLVWK (Leu, 68%)	DAFYLGMMCK (Alanine)	
Module 2	DASTVAAVCK (Tyr, 91%)	DASTVAAVCK (Tyr, 91%)	DASTVAAVCK (Tyr, 91%)	DASTVAAVCK (Tyr, 91%)	DASTVAAVCK (Tyr, 94%)	DASTVAAVCK (Tyr, 94%)	DASTVAAVCK (Tyrosine)	
Module 3	DVLLVGTIAK (Leu, 62%)	DVLLVGTIAK (Leu, 62%)	DVLL-----K (?)	DVLLVGTIAK (Leu, 62%)	DVQLMGSIAK (Glu, 38%)	DVLLVGTIAK (Leu, 62%)	DVLLVGTIAK (Glutamic acid)	
	MFI8933342	WP_138963347	WP_304459971	WP_278194070	WP_391632539	WP_345386641	Pek20	
Module 4	DIFHLGLLCK (Hpg, 100%)	DIFHLGLLCK (Hpg, 97%)	DIFHLGLLCK (Hpg, 97%)	DIFHLGLLCK (Hpg, 97%)	DIFHLGLLCK (Hpg, 100%)	DIFHLGLLCK (Hpg, 100%)	DIFHLGLLCK (Hpg)	
Module 5	DAVHLGLLCK (Hpg, 97%)	DAVHLGLLCK (Hpg, 94%)	DAVHLGLLCK (Hpg, 97%)	DAVHLGLLCK (Hpg, 97%)	DAVHLGLLCK (Hpg, 97%)	DAVHLGLLCK (Hpg, 100%)	DAVHLGLLCK (Hpg)	
Module 6	DASTLGAICK (Bht, 100%)	DASTLGAICK (Bht, 100%)	DASTLGAICK (Bht, 100%)	DASTLGAICK (Bht, 100%)	DASTLGAICK (Bht, 100%)	DASTLGAICK (Bht, 100%)	DASTLGAICK (Bht)	
	MFI8933343	WP_269078107	WP_304459970	WP_308402225	WP_391638893	WP_345386639	Pek21	
Module 7	DPYHGGTLCK (Dpg, 100%)	DPYHGGTLCK (Dpg, 100%)	DPYHGGTLCK (Dpg, 100%)	DPYHGGTLCK (Dpg, 100%)	DPYHGGTLCK (Dpg, 100%)	DPYHGGTLCK (Dpg, 100%)	DPYHGGTLCK (Dpg)	
StrR	MFI8933329	WP_138963327	WP_369696689	WP_242714316	WP_391632549	WP_345386593	Pek7	
ABC	MFI8933339	WP_249402009	WP_304459975	WP_242728487	WP_391632541	WP_345386645	Pek17	
OxyA	MFI8933345	WP_249402011	WP_304459968.1	WP_242714304	WP_391632536	WP_345386635	Pek23	
OxyB	MFI8933346	WP_138963353	WP_304459967	WP_242714303	WP_391632535	WP_345386633	Pek24	
OxyC	MFI8933347	WP_138963355	WP_304459966	WP_242714302	WP_391632534	WP_345386631	Pek26	
Halogenase	MFI8933348	WP_138963357	WP_304459965	WP_242714301	WP_391632533	WP_345386629	Pek27	
N-methyltransferase	MFI8933350	WP_138963361	WP_304459963	WP_242714299	WP_391632531	WP_345386625	Pek30	
Aba23 GT1	MFI8933349	WP_138963359	WP_304459964	WP_242714300	WP_391632532	WP_345386627		

	(1)	(2)	(3)	(4)	(5)	(6)	(7)	(8)
Dahp						WP_345386599		
Pdh	MFI8933341	WP_269078106	WP_304459972	WP_374120573	WP_391632526	WP_345386647	Pek19	
DpgA	MFI8933358	WP_138963372	WP_304459955	WP_242714292	WP_391632557	WP_345386605	Pek37	
DpgB	MFI8933357	WP_249402014	WP_304459956	WP_242714293	WP_391632558	WP_345386607	Pek38	
DpgC	MFI8933359	WP_138963374	WP_304459953	WP_242714291	WP_391632556	WP_345386603	Pek39	
DpgD						WP_345386601		
HpgT	MFI8933351	WP_249402012	WP_304459962	WP_242728485	WP_391632530	WP_345386623	Pek31	
Hmo	MFI8933356	WP_138963369	WP_304459957	WP_242714294	WP_391632527	WP_345386614	Pek36	
HmaS	MFI8933355	WP_249402013	WP_304465416	WP_242714295	WP_391632568	WP_345386816	Pek35	
Bhp	MFI8933352	WP_346347461	WP_369696686	WP_242714298	WP_391632529	WP_345386620	Pek32	
OxyD	MFI8933354	WP_138963367	WP_304459958	WP_242714296	WP_391632528	WP_345386616	Pek34	
BpsD	MFI8933353	WP_138963365	WP_304459959	WP_242714297		WP_345386618	Pek33	
VanK	MFI8933361	WP_346347407	WP_304459951	WP_374120571	WP_391632560			
					WP_391632565			StaK
					WP_391632564			StaL
					WP_391632563			StaM
					WP_391632562			StaN
					WP_391632561			VanH
					WP_391632560			StaO
					WP_391632559			StaP

Supplementary Figures

```
AMYBAR_000817       TAATCTGTCCTGTACTTTGGGATAAGCCCTGGAAACGGGGTCTAATACCGAATATGACCA 180
AMYBAR_001096       TAATCTGTCCTGTACTTTGGGATAAGCCCTGGAAACGGGGTCTAATACCGAATATGACCA 180
AMYBAR_002252       TAATCTGTCCTGTACTTTGGGATAAGCCCTGGAAACGGGGTCTAATACCGAATATGACTT 180
                    **********************************************************

AMYBAR_000817       TCTCAGGCATCTGGGGTGGTGGAAAGTTCTGGCGGTACAGGGTGAGCCCGCGGCCTATCA 240
AMYBAR_001096       TCTCAGGCATCTGGGGTGGTGGAAAGTTCTGGCGGTACAGGGTGAGCCCGCGGCCTATCA 240
AMYBAR_002252       CGCGAGGCATCTCGTGGGGTGGAAAGTTCTGGCGGTACAGGGTGAGCCCGCGGCCTATCA 240
                        ******** * * *******************************************

AMYBAR_000817       GCTTGTTGGTGGGGTGATGGCCTACCAAGGCGACGACGGGTAGCCGGCCTGAGAGGGTGA 300
AMYBAR_001096       GCTTGTTGGTGGGGTGATGGCCTACCAAGGCGACGACGGGTAGCCGGCCTGAGAGGGTGA 300
AMYBAR_002252       GCTTGTTGGTGGGGTGATGGCCTACCAAGGCGACGACGGGTAGCCGGCCTGAGAGGGTGA 300
                    ************************************************************
```

Figure S1. Multiple nucleic acid sequence alignment of 121-300 bp region of *A. bartoniae* DSM 45807 16S rRNA genes showing sequence microheterogeneity. Alignment was generated using Clustal Omega [8].

(a)

	Non-ribosomal code	Substrate specificity
Module 1	DAFHLGLLCK	Hpg (100% match)
Module 2	DTSKVAAICK	Bht (100% match)
Module 3	DPFNQGTFCK	Dpg (90% match)
Module 4	DIFHLGLLCK	Hpg (100% match)
Module 5	DAYHLGLLCK	Hpg (94% match)
Module 6	DASTLGAICK	Bht (100% match)
Module 7	DPYHGGTLCK	Dpg (90% match)

(b)

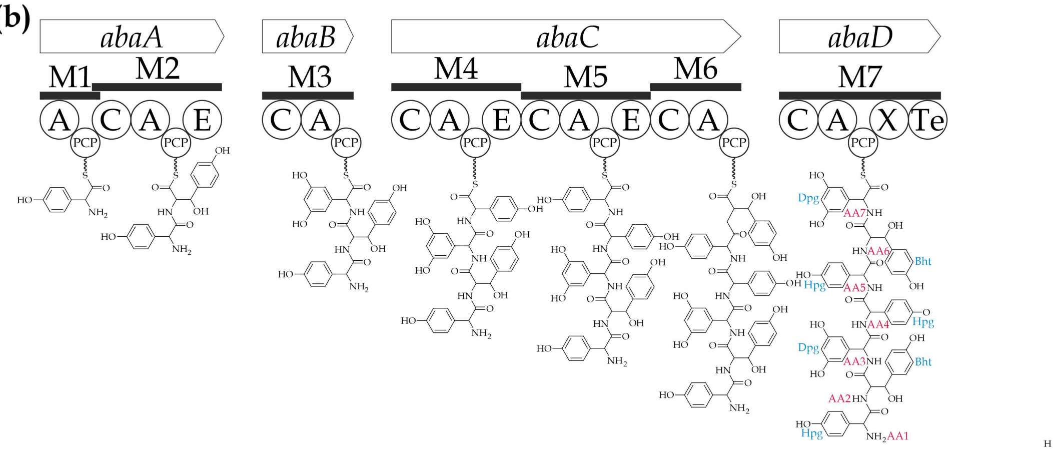

Figure S2. (a) substrate specificity of A-domains of *A. bartoniae* GPA NRPS (non-ribosomal codes were obtained from antiSMASH [7] analysis output) and (**b**) the organization of GPA NRPS assembly line in *A. bartoniae.*

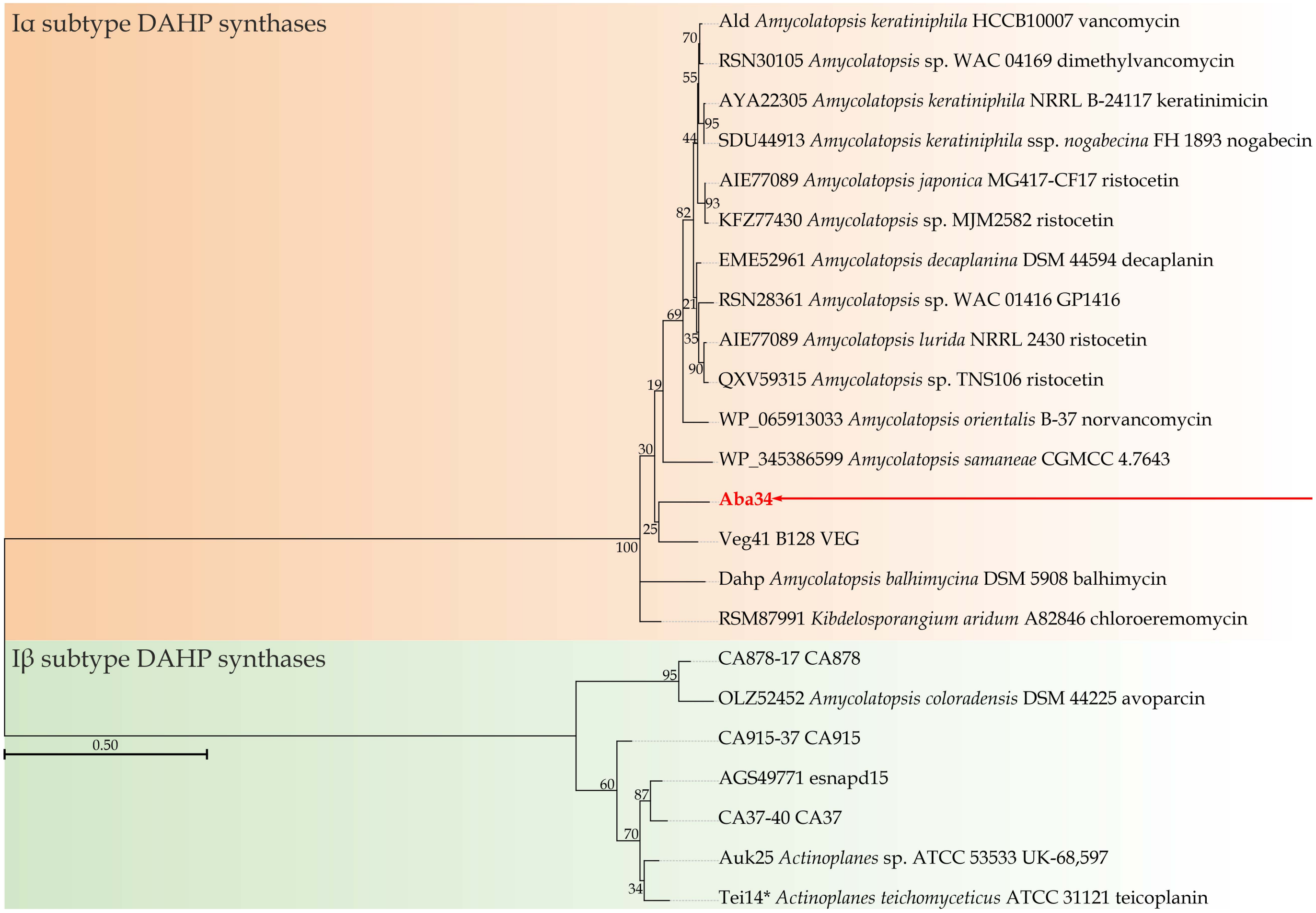

Figure S3. Phylogeny of 23 DAHP synthases (including *A. bartoniae* Aba34) coded in various GPA BGCs. Phylogenetic tree was inferred by using the Maximum Likelihood method and JTT matrix-based model [9] with a discrete Gamma distribution to model evolutionary rate differences among sites (3 categories). Bootstrap values (n=500) are indicated at the nodes. The tree is drawn to scale; scale bar represents the number of nucleotide substitutions per site. *aba*-encoded DAHP synthase is highlighted with a red arrow.

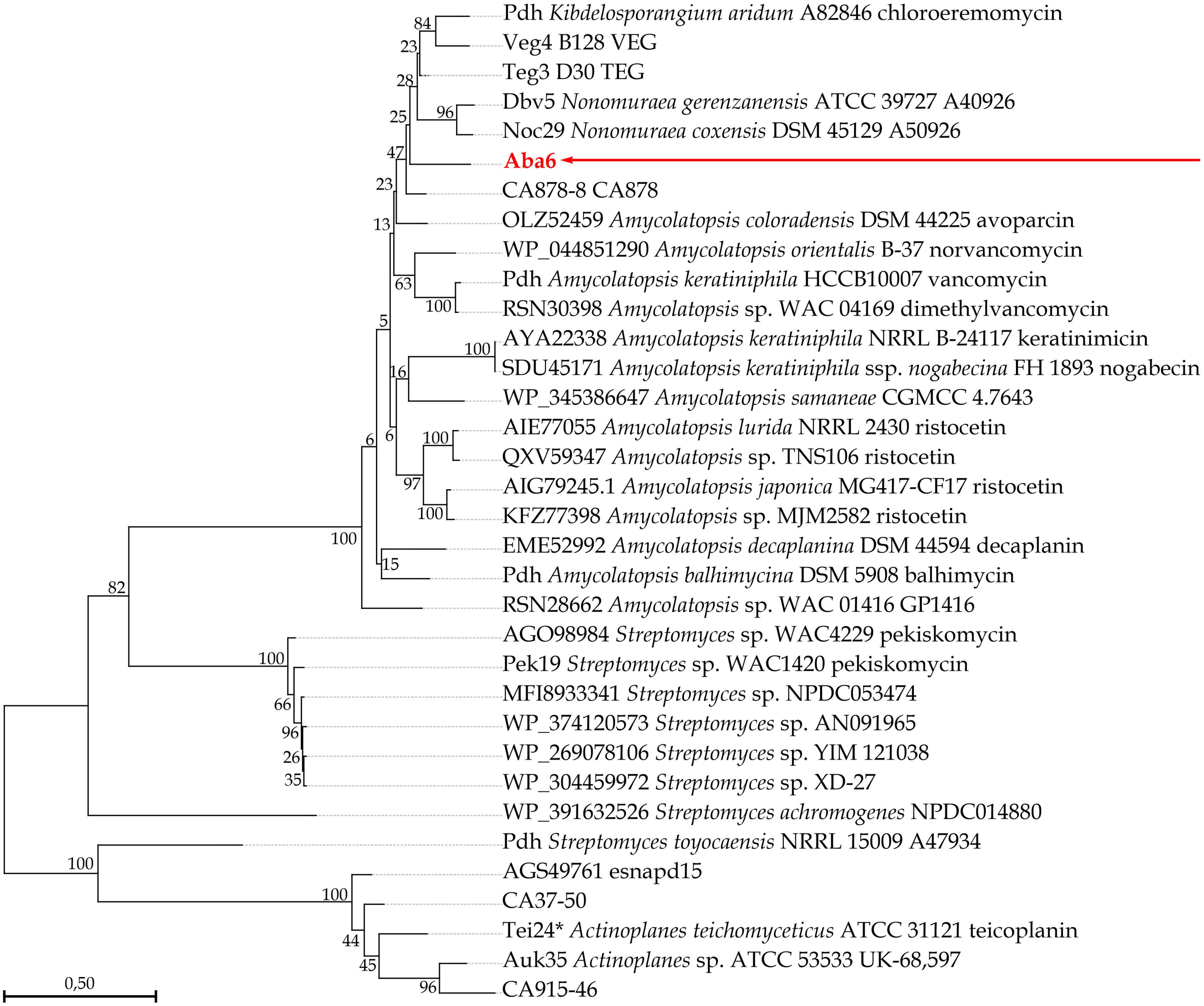

Figure S4. Phylogeny of 34 PDHs (including *A. bartoniae* Aba6) coded in various GPA BGCs. Phylogenetic tree was inferred by using the Maximum Likelihood method and JTT matrix-based model [9] with a discrete Gamma distribution to model evolutionary rate differences among sites (3 categories). Bootstrap values (n=500) are indicated at the nodes. The tree is drawn to scale; scale bar represents the number of nucleotide substitutions per site. *aba*-encoded PDH is highlighted with a red arrow.

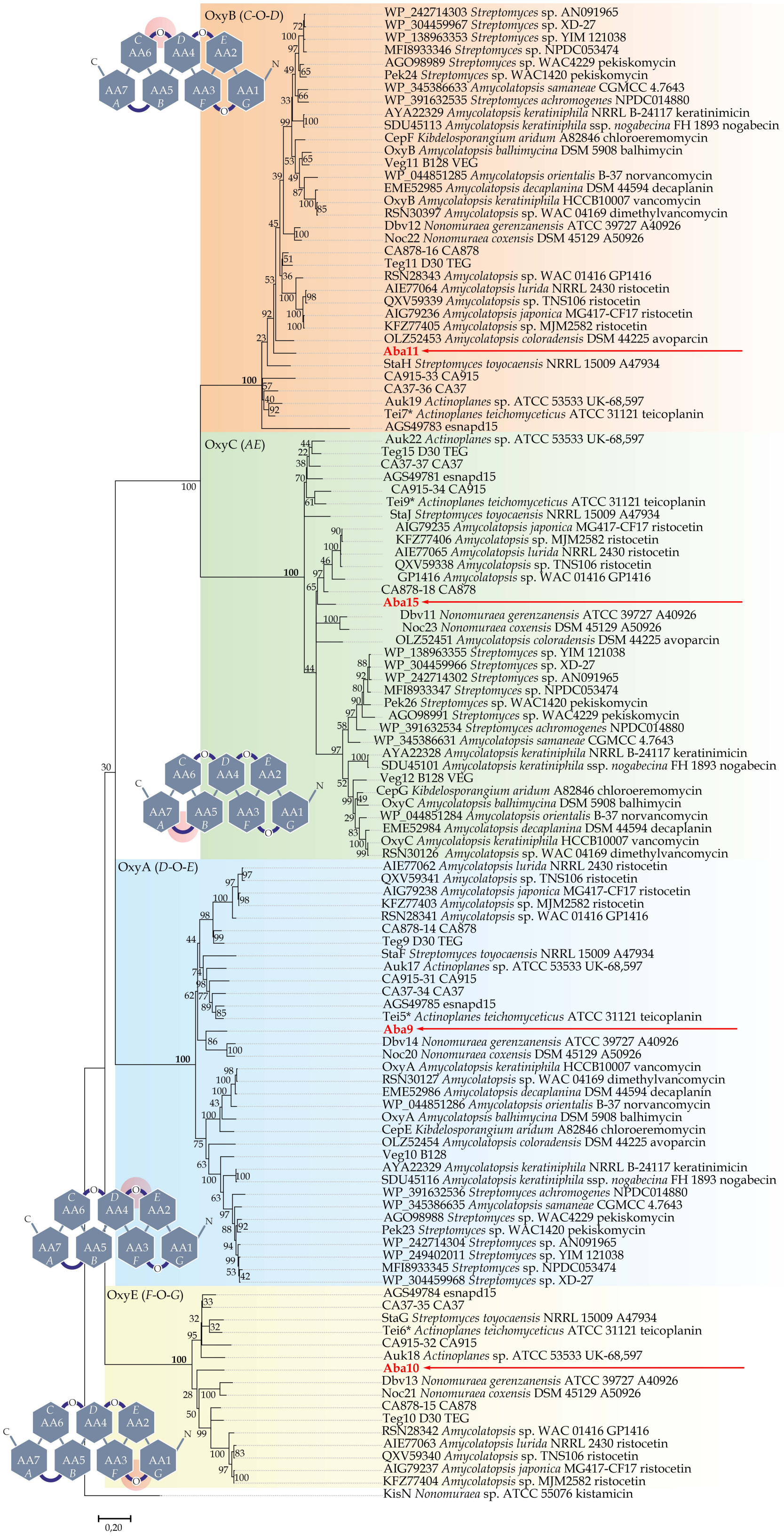

Figure S5. Phylogeny of 119 cross-linking monooxygenases (including *A. bartoniae* Aba9, Aba10, Aba11, and Aba15) coded in various GPA BGCs. KisN coded in kistamicin BGC from *Nonomuraea* sp. ATCC 55076 was used as an outgroup. Phylogenetic tree was inferred by using the Maximum Likelihood method and JTT matrix-based model [9] with a discrete Gamma distribution to model evolutionary rate differences among sites (3 categories); the rate variation model allowed for some sites to be evolutionarily invariable. Bootstrap values (n=500) are indicated at the nodes. The tree is drawn to scale; scale bar represents the number of nucleotide substitutions per site. *aba*-encoded cross-linking monooxygenases are marked with red arrows.

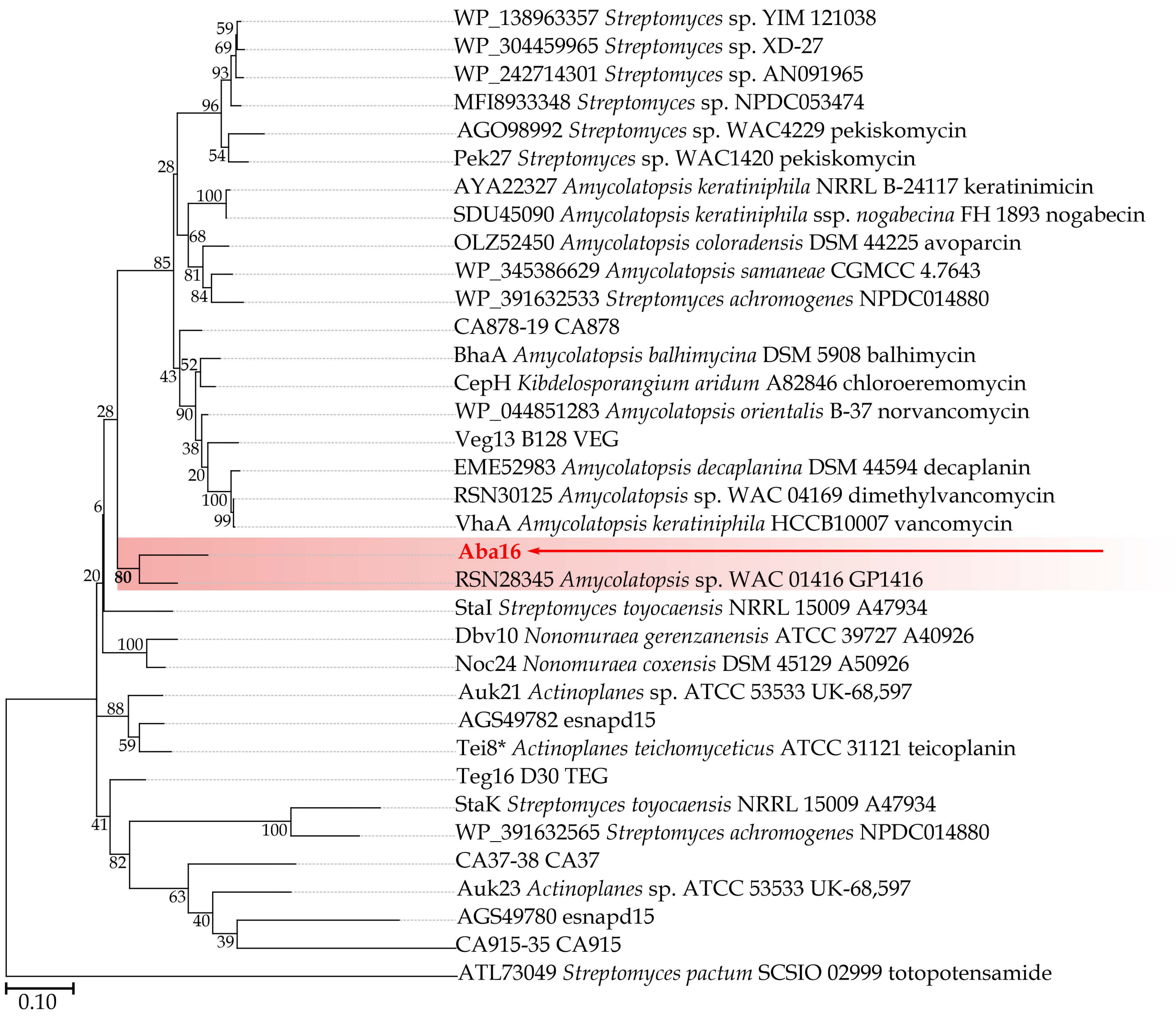

Figure S6. Phylogeny of 34 halogenases (including *A. bartoniae* Aba16) coded in various GPA BGCs and ATL73049 from *Streptomyces pactum* SCSIO 02999 totopotensamide BGC (used as an outgroup). Phylogenetic tree was inferred by using the Maximum Likelihood method and JTT matrix-based model [9] with a discrete Gamma distribution to model evolutionary rate differences among sites (3 categories). Bootstrap values (n=500) are indicated at the nodes. The tree is drawn to scale; scale bar represents the number of nucleotide substitutions per site. *aba*-encoded halogenase is marked with a red arrow and the corresponding clade of Aba16 and RSN28345 is highlighted in red.

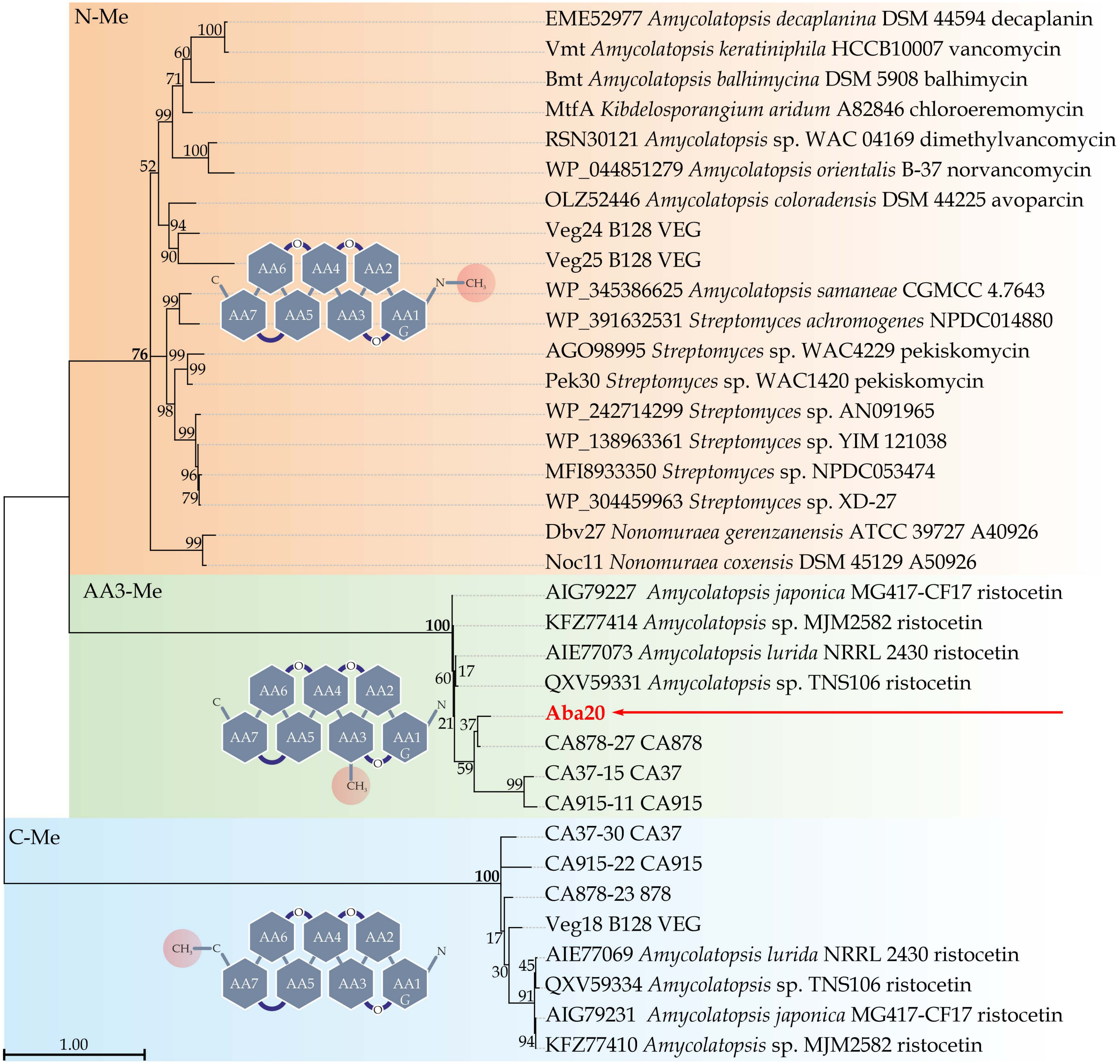

Figure S7. Phylogeny of 35 methyltransferases (including *A. bartoniae* Aba20) coded in various GPA BGCs. Phylogenetic tree was inferred by using the Maximum Likelihood method and JTT matrix-based model [9] with a discrete Gamma distribution to model evolutionary rate differences among sites (3 categories). Bootstrap values (n=500) are indicated at the nodes. The tree is drawn to scale; scale bar represents the number of nucleotide substitutions per site. *aba*-encoded methyltransferase is marked with a red arrow; main clades were highlighted with different colors according to the substrate specificity of the enzymes.

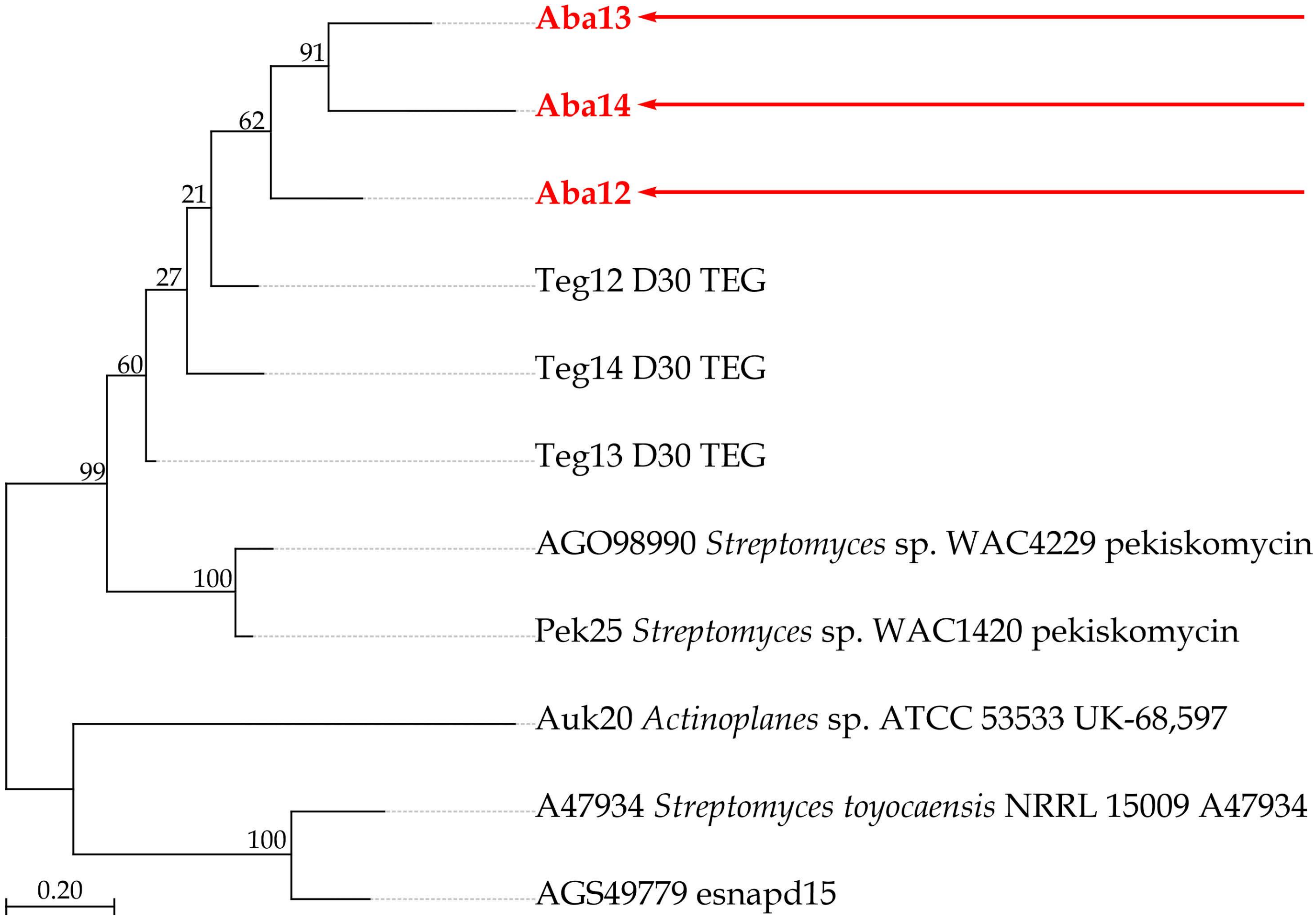

Figure S8. Phylogeny of 11 sulfotransferases (including *A. bartoniae* Aba12, Aba13, and Aba14) coded in various GPA BGCs. Phylogenetic tree was inferred by using the Maximum Likelihood method and JTT matrix-based model [9] with a discrete Gamma distribution to model evolutionary rate differences among sites (3 categories). Bootstrap values (n=500) are indicated at the nodes. The tree is drawn to scale; scale bar represents the number of nucleotide substitutions per site. *aba*-encoded sulfotransferases are marked with red arrows.

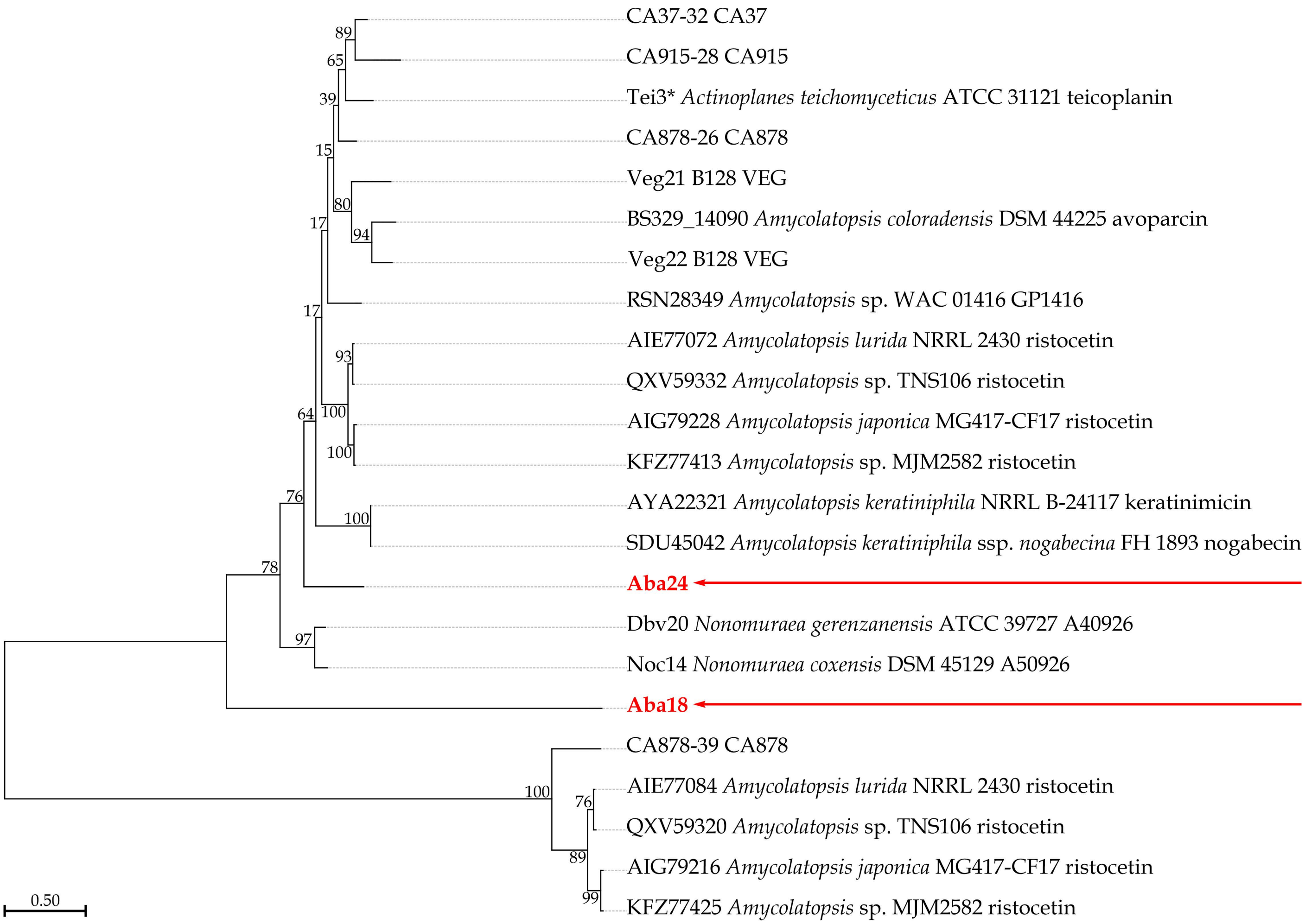

Figure S9. Phylogeny of 23 GT39-GTFs (including *A. bartoniae* Aba18 and Aba24) coded in various GPA BGCs. Phylogenetic tree was inferred by using the Maximum Likelihood method and Whelan and Goldman + Freq. model [10] with a discrete Gamma distribution to model evolutionary rate differences among sites (3 categories). Bootstrap values (n=500) are indicated at the nodes. The tree is drawn to scale; scale bar represents the number of nucleotide substitutions per site. *aba*-encoded GT39-GTFs are marked with red arrows.

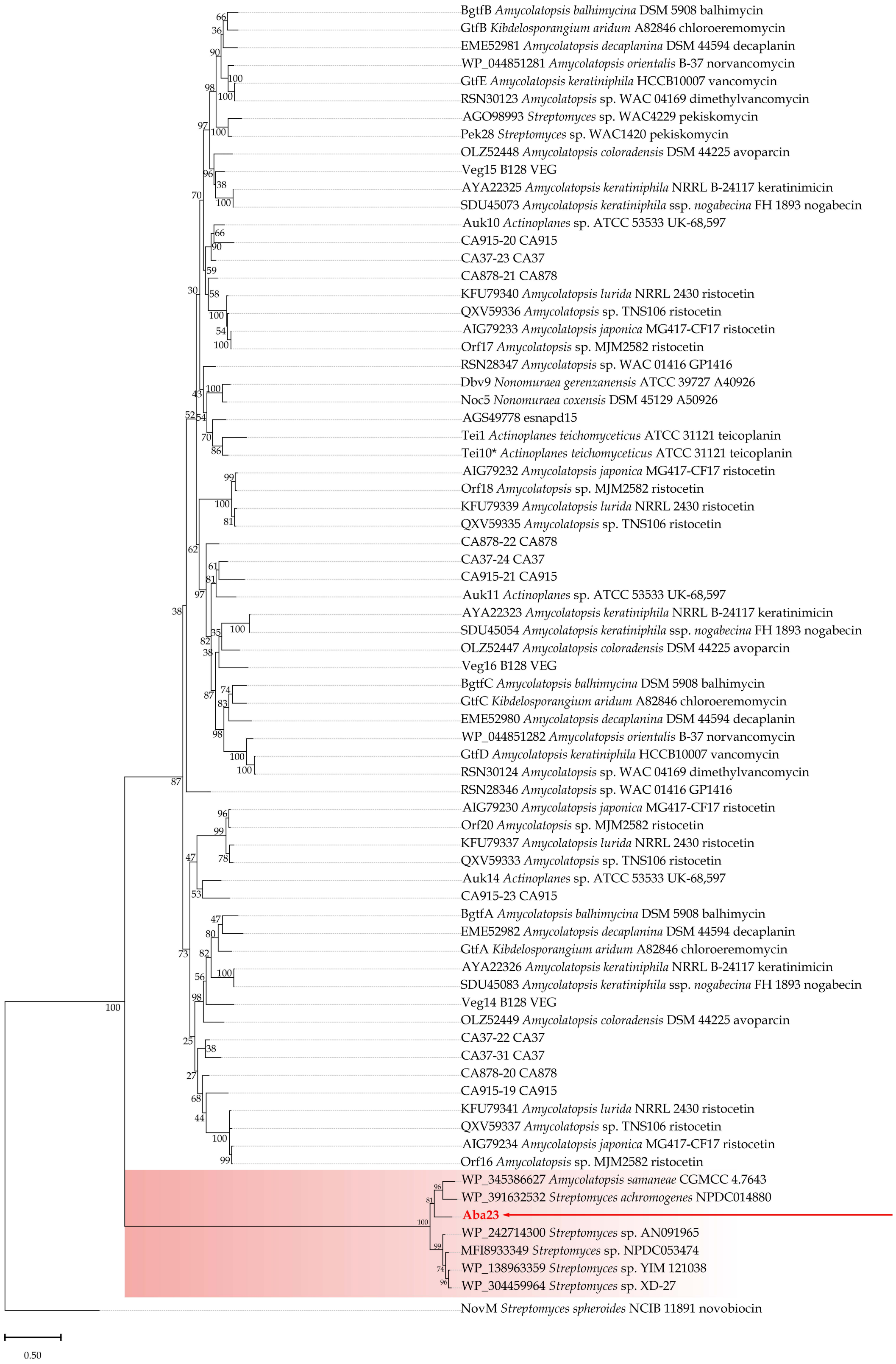

Figure S10. Phylogeny of 76 GT1-GTFs (including *A. bartoniae* Aba23) coded in various GPA BGCs and NovM from *Streptomyces spheroides* NCIB 11891 novobiocin BGC (used as an outgroup). Phylogenetic tree was inferred by using the Maximum Likelihood method and JTT matrix-based model [9] with a discrete Gamma distribution to model evolutionary rate differences among sites (3 categories); the rate variation model allowed for some sites to be evolutionarily invariable. Bootstrap values (n=500) are indicated at the nodes. The tree is drawn to scale; scale bar represents the number of nucleotide substitutions per site. *aba*-encoded GT1-GTF is marked with red arrows, the clade containing its orthologues from newly identified GPA BGCs is highlighted in red.

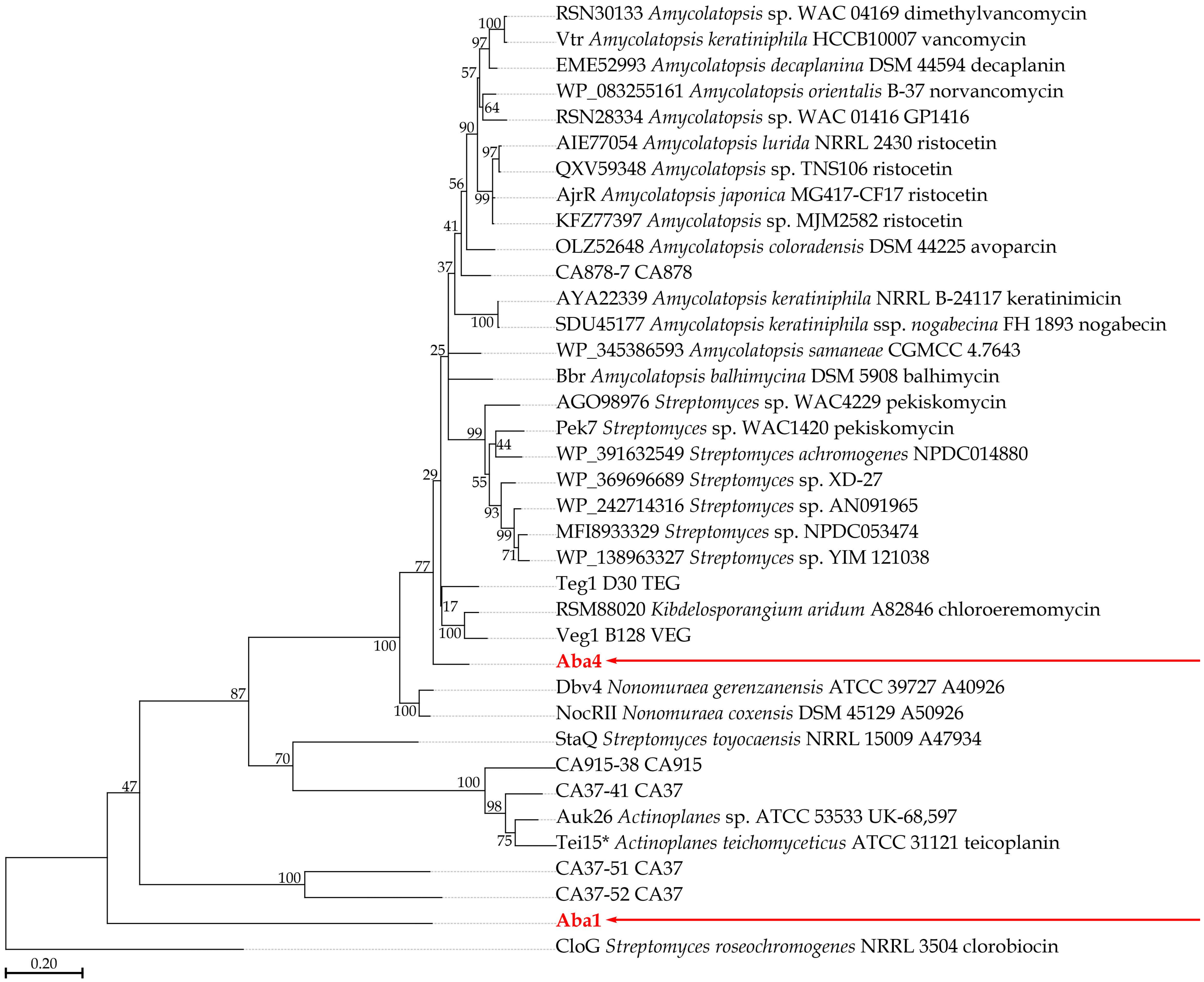

Figure S11. Phylogeny of 36 StrR-like transcriptional regulators coded in various GPA BGCs and CloG from *Streptomyces roseochromogenes* NRRL 3504 clorobiocin BGC (used as an outgroup). Phylogenetic tree was inferred by using the Maximum Likelihood method and JTT matrix-based model [9] with a discrete Gamma distribution to model evolutionary rate differences among sites (3 categories); the rate variation model allowed for some sites to be evolutionarily invariable. Bootstrap values (n=500) are indicated at the nodes. The tree is drawn to scale; scale bar represents the number of nucleotide substitutions per site. *aba*-encoded StrR-regulators are marked with red arrows.

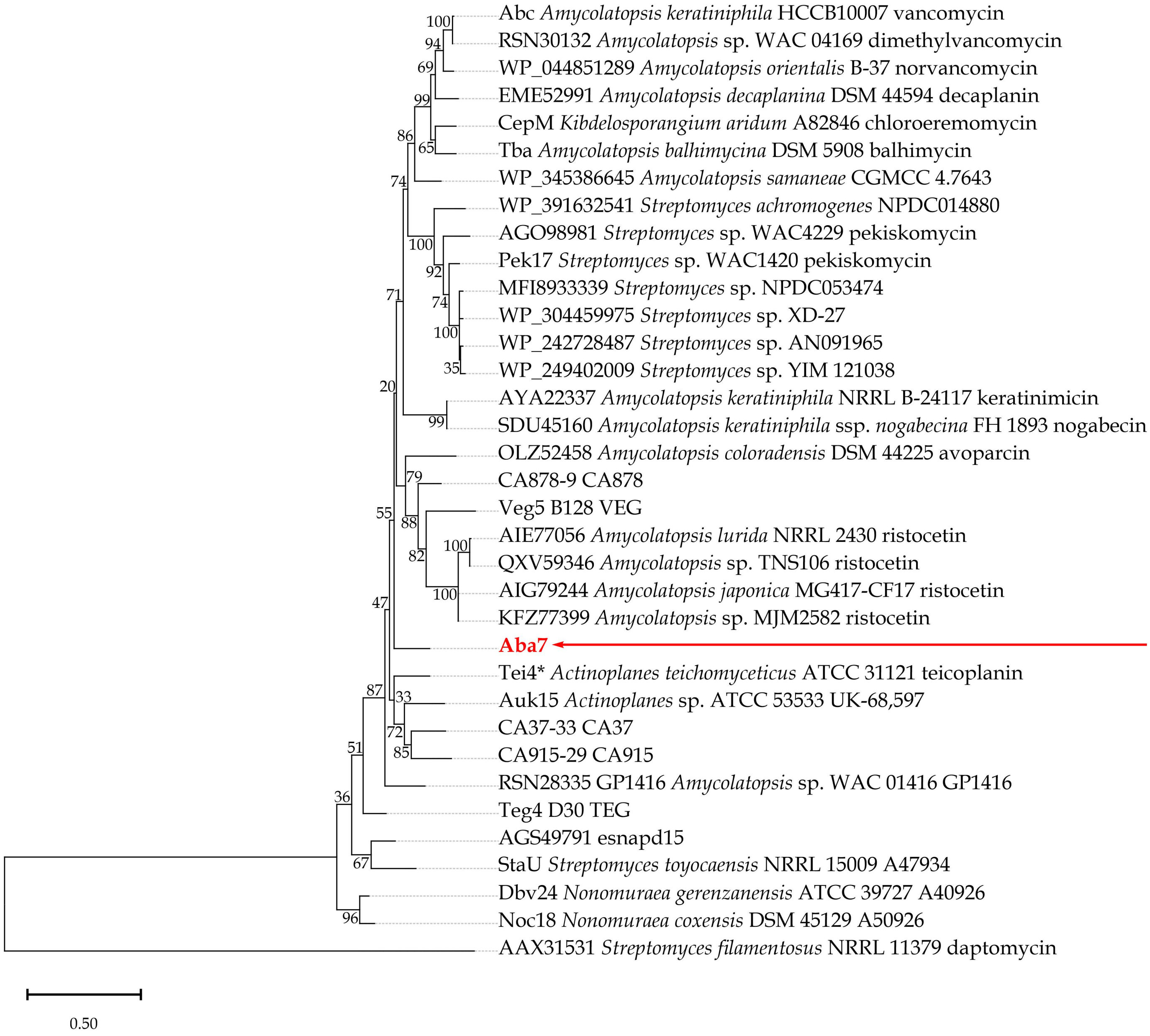

Figure S12. Phylogeny of 34 ABC-transporters coded in various GPA BGCs and AAX31531 from *Streptomyces filamentosus* NRRL 11379 daptomycin BGC (used as an outgroup). Phylogenetic tree was inferred by using the Maximum Likelihood method and JTT matrix-based model [9] with a discrete Gamma distribution to model evolutionary rate differences among sites (3 categories). Bootstrap values (n=500) are indicated at the nodes. The tree is drawn to scale; scale bar represents the number of nucleotide substitutions per site. *aba*-encoded ABC-transporter is marked with red arrow.

(a)

	Streptomyces sp. NPDC053474	*Streptomyces* sp. YIM 121038	*Streptomyces* sp. XD-27	*Streptomyces* sp. AN091965	*Streptomyces achromogenes* NPDC014880	*Amycolatopsis samaneae* CGMCC 4.7643	*Streptomyces* sp. WAC1420
Module 1	DAFYQGLVWK (Leu, 68%)	DAFYQGLVWK (Leu, 68%)	DAFYQGLVWK (Leu, 68%)	DAFYQGLVWK (Leu, 68%)	DAFYQGLVWK (Leu, 65%)	DAFYQGLVWK (Leu, 68%)	DAFYLGMMCK Alanine
Module 2	DASTVAAVCK (Tyr, 91%)	DASTVAAVCK (Tyr, 91%)	DASTVAAVCK (Tyr, 91%)	DASTVAAVCK (Tyr, 91%)	DASTVAAVCK (Tyr, 94%)	DASTVAAVCK (Tyr, 94%)	DASTVAAVCK Tyrosine
Module 3	DVLLVGTIAK (Leu, 62%)	DVLLVGTIAK (Leu, 62%)	DVLL-----K (?)	DVLLVGTIAK (Leu, 62%)	DVQLMGSIAK (Glu, 38%)	DVLLVGTIAK (Leu, 62%)	DVLLVGTIAK Glutamic acid
Module 4	DIFHLGLLCK (Hpg, 100%)	DIFHLGLLCK (Hpg, 97%)	DIFHLGLLCK (Hpg, 97%)	DIFHLGLLCK (Hpg, 97%)	DIFHLGLLCK (Hpg, 100%)	DIFHLGLLCK (Hpg, 100%)	DIFHLGLLCK Hpg
Module 5	DAVHLGLLCK (Hpg, 97%)	DAVHLGLLCK (Hpg, 94%)	DAVHLGLLCK (Hpg, 97%)	DAVHLGLLCK (Hpg, 97%)	DAVHLGLLCK (Hpg, 97%)	DAVHLGLLCK (Hpg, 100%)	DAVHLGLLCK Hpg
Module 6	DASTLGAICK (Bht, 100%)	DASTLGAICK (Bht, 100%)	DASTLGAICK (Bht, 100%)	DASTLGAICK (Bht, 100%)	DASTLGAICK (Bht, 100%)	DASTLGAICK (Bht, 100%)	DASTLGAICK Bht
Module 7	DPYHGGTLCK (Dpg, 100%)	DPYHGGTLCK (Dpg, 100%)	DPYHGGTLCK (Dpg, 100%)	DPYHGGTLCK (Dpg, 100%)	DPYHGGTLCK (Dpg, 100%)	DPYHGGTLCK (Dpg, 100%)	DPYHGGTLCK Dpg

(b)

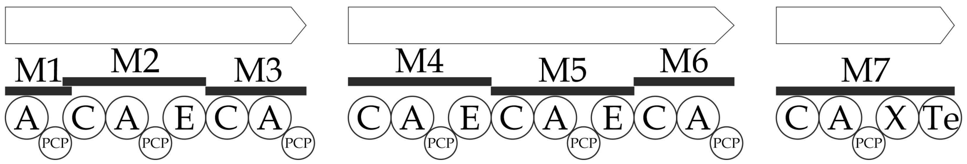

Figure S13. (a) substrate specificity of A-domains of NRPS coded in a set of 6 newly discovered GPA BGCs and *pek* BGC (for comparison) (non-ribosomal codes were obtained from antiSMASH [7] analysis output) and (**b**) the organization of corresponding NRPSs (identical in all 6 GPA BGCs).

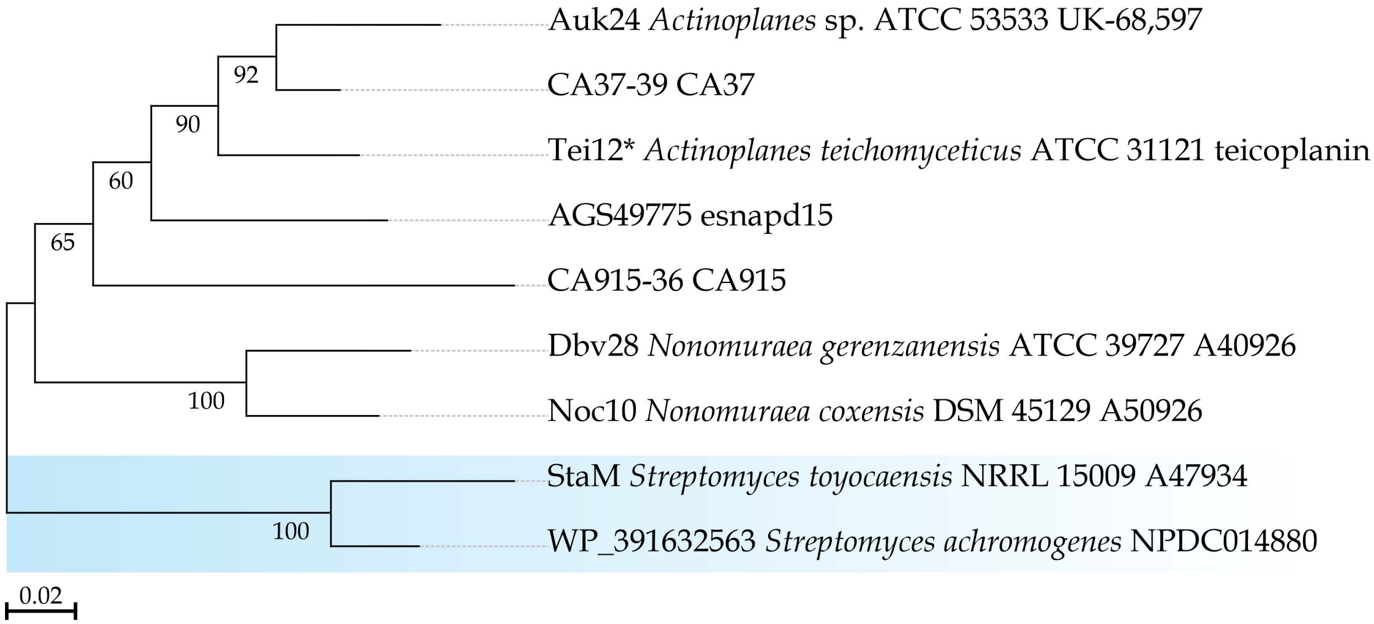

Figure S14. Phylogeny of 9 β-hydroxylases coded in various GPA BGCs. Phylogenetic tree was inferred by using the Maximum Likelihood method and JTT matrix-based model [9] with a discrete Gamma distribution to model evolutionary rate differences among sites (3 categories). Bootstrap values (n=500) are indicated at the nodes. The tree is drawn to scale; scale bar represents the number of nucleotide substitutions per site. StaM and its homologue from NPDC014880 BGC belong to one clade (highlighted in blue).

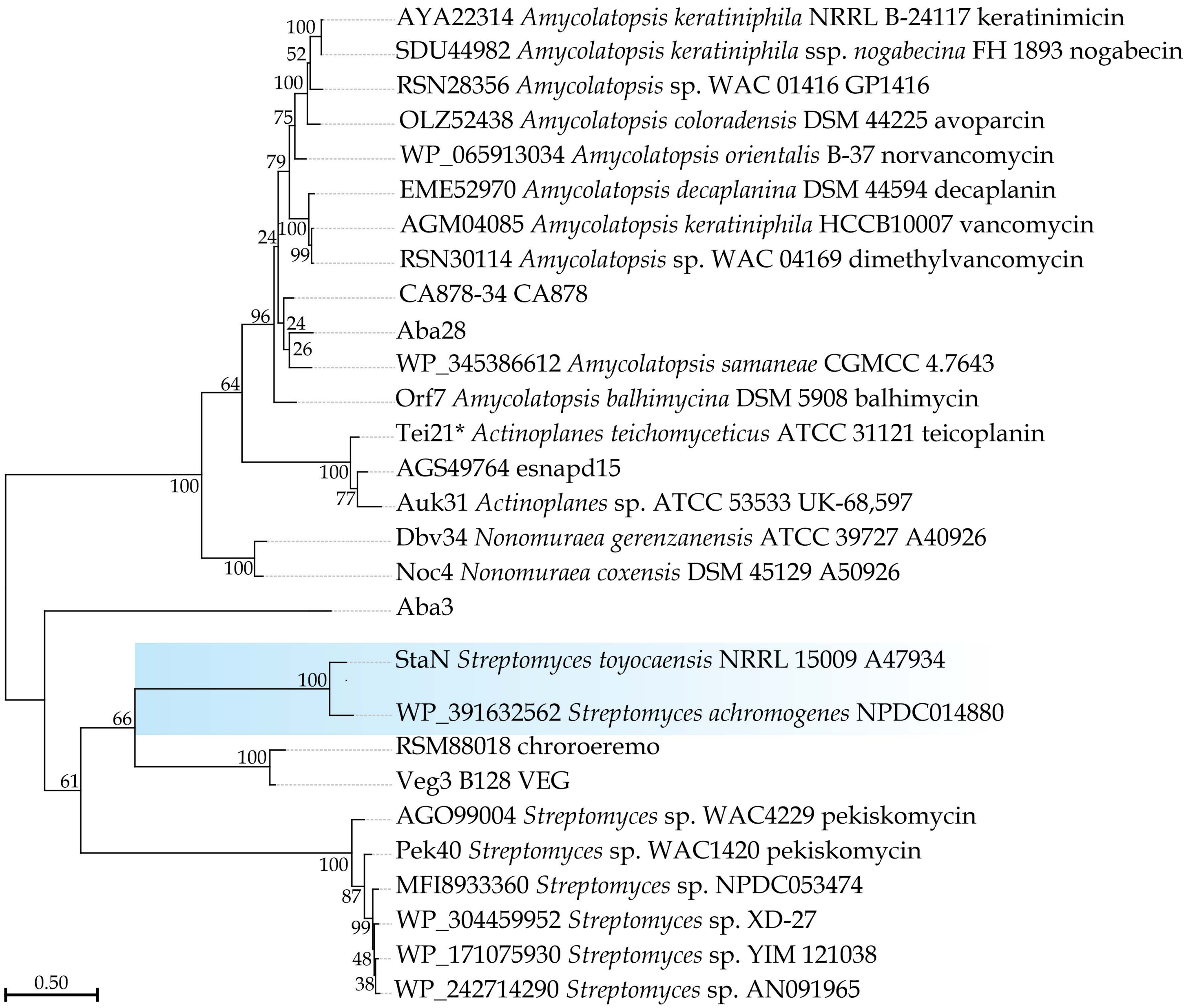

Figure S15. Phylogeny of 29 membrane ion antiporters s coded in various GPA BGCs. Phylogenetic tree was inferred by using the Maximum Likelihood method and Le_Gascuel_2008 model [11] with a discrete Gamma distribution to model evolutionary rate differences among sites (3 categories). Bootstrap values (n=500) are indicated at the nodes. The tree is drawn to scale; scale bar represents the number of nucleotide substitutions per site. StaN and its homologue from NPDC014880 BGC belong to one clade (highlighted in blue).

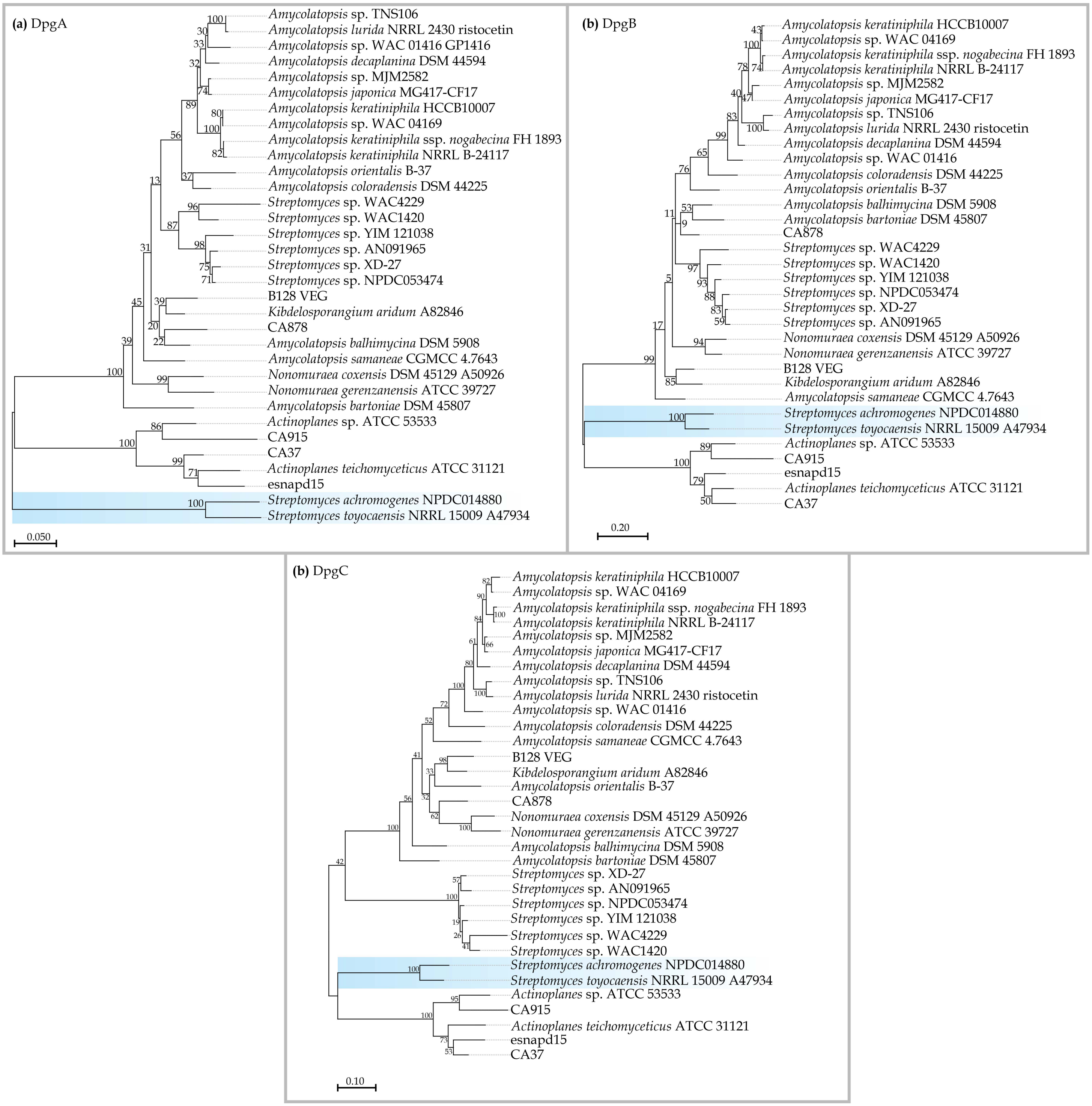

Figure S16. Phylogeny of 33 DpgA **(a)**, 33 DpgB **(b)**, and 33 DpgC **(c)** proteins coded in various GPA BGCs. Phylogenetic trees were inferred by using the Maximum Likelihood method and JTT matrix-based model [9] with a discrete Gamma distribution to model evolutionary rate differences among sites (3 categories). Bootstrap values (n=500) are indicated at the nodes. Trees are drawn to scale; scale bar represents the number of nucleotide substitutions per site. DpgA, DpgB, and DpgC coded in *S. toyocaensis* NRRL 15009 A47934 BGC and counterparts from NPDC014880 BGC group together (highlighted in blue).

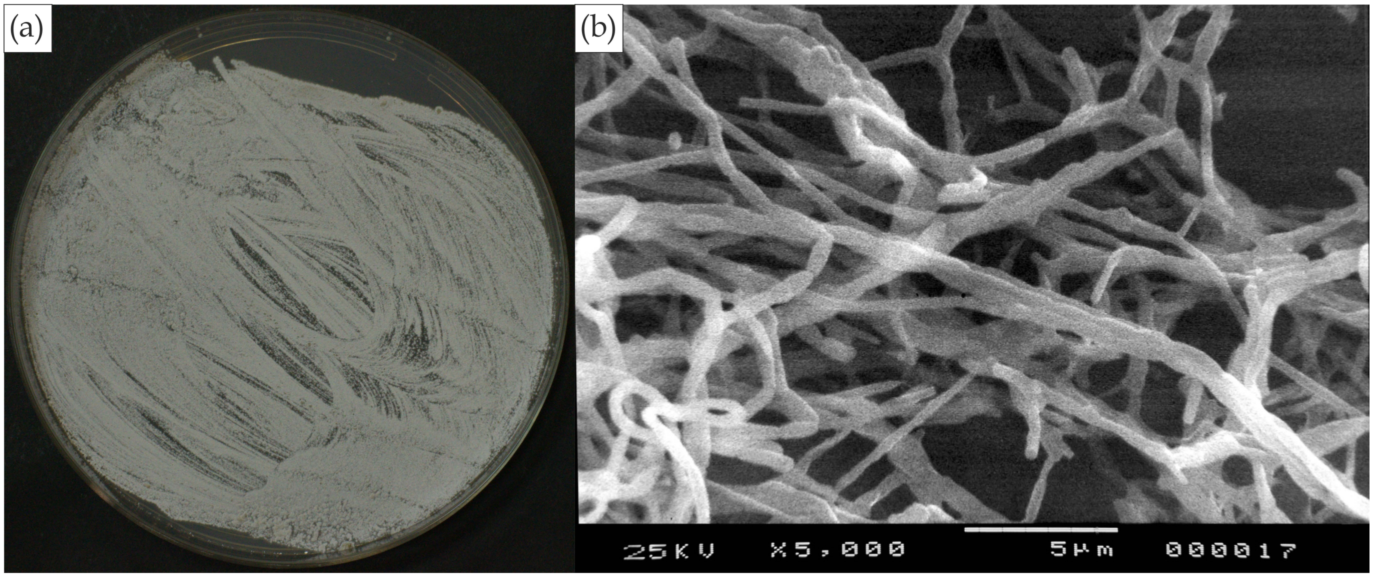

Figure S17. A typical aspect of *A. bartoniae* DSM 45807 lawn after 120 h of cultivation on ISP5 **(a)** that supports abundant sporulation **(b)**.

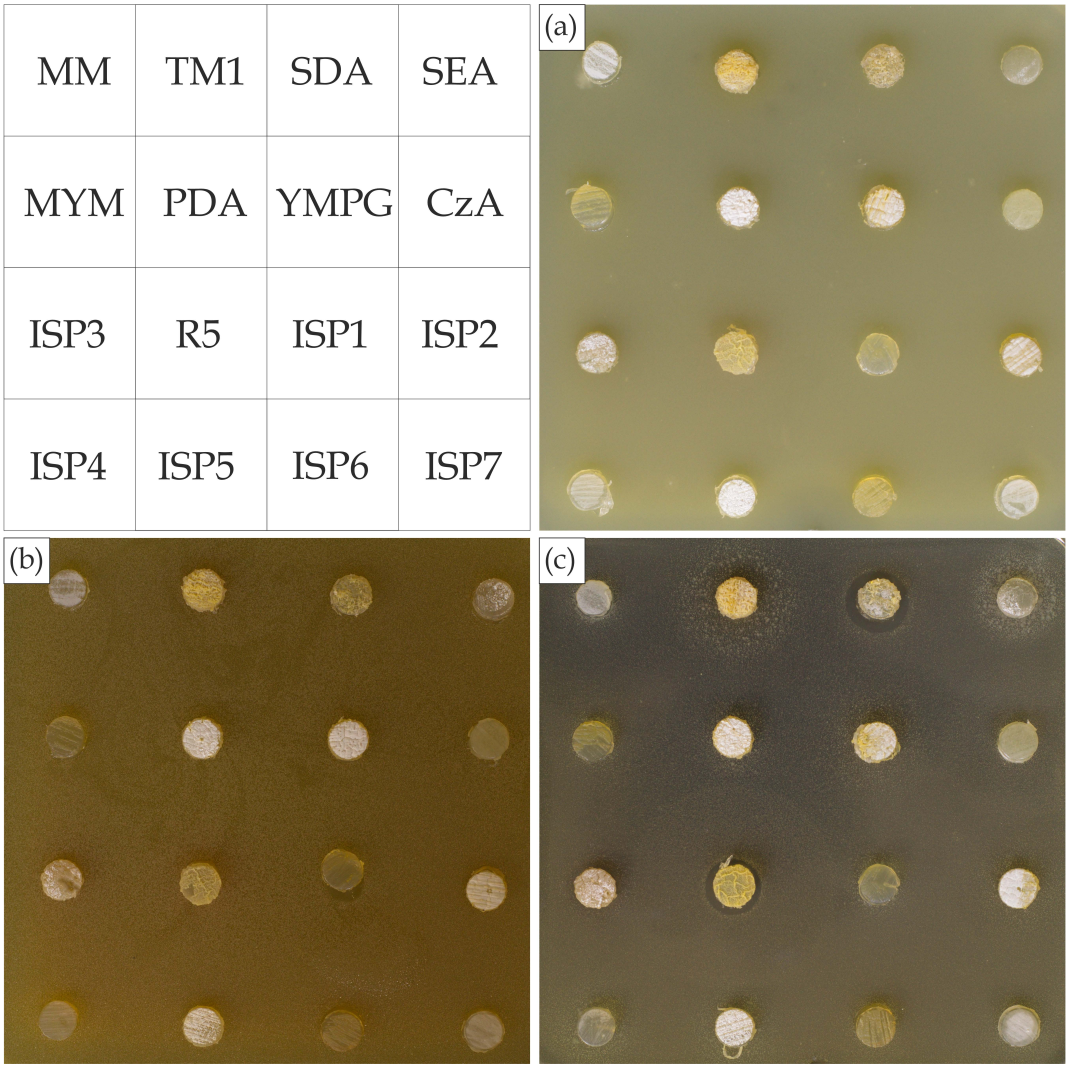

Figure S18. *E. coli* DH5α **(a)**, *D. hansenii* VKM Y-9 **(b)**, and *B. subtilis* HB0950 **(c)** growth inhibition assays using agar plugs taken from 120 h old lawns *A. bartoniae* DSM 45807 cultivated on 16 various solid media (please refer to the legend).

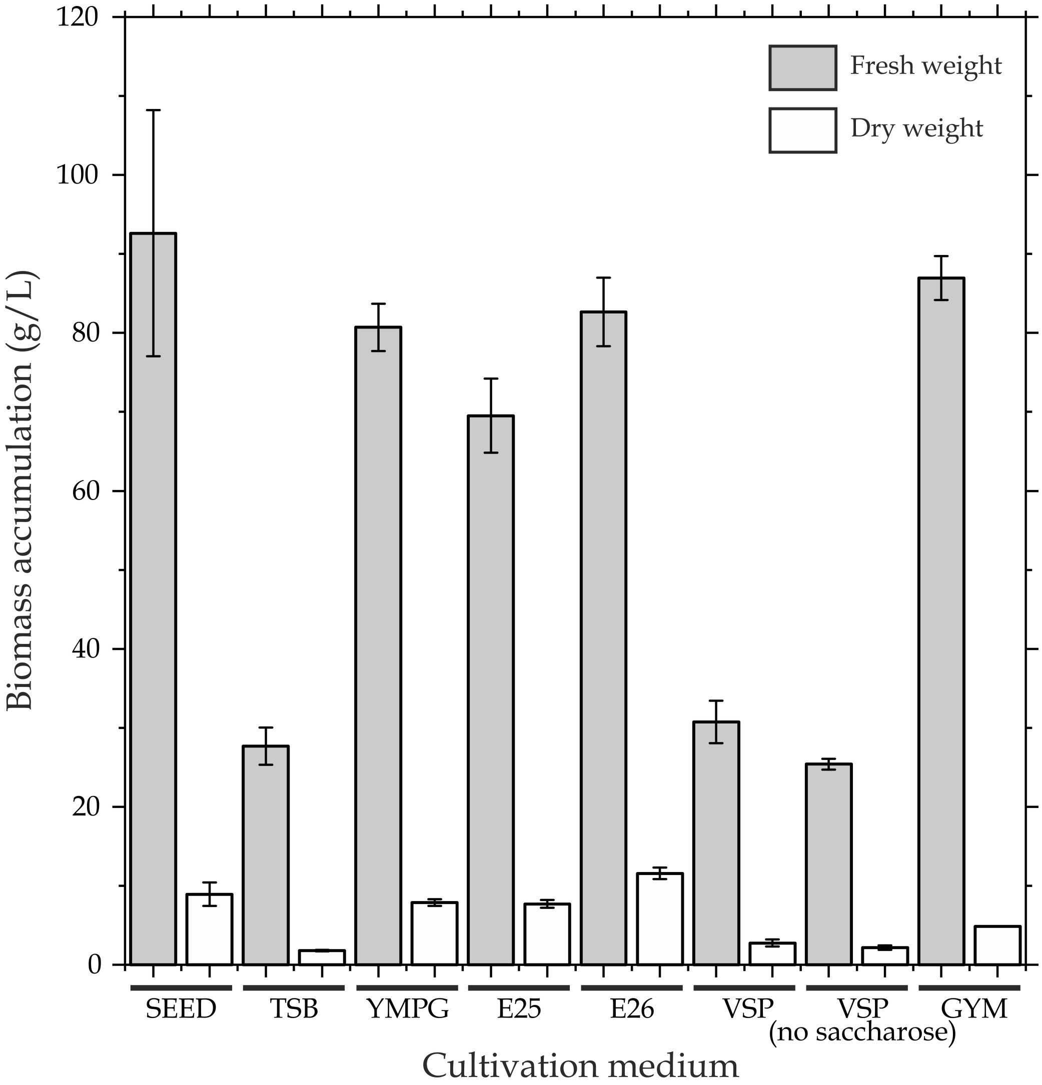

Figure S19. *A. bartoniae* DSM 45807 biomass accumulation in various liquid media after 72 h of cultivation. Data represent mean values of at least three independent experiments ± 2SD.

(a)

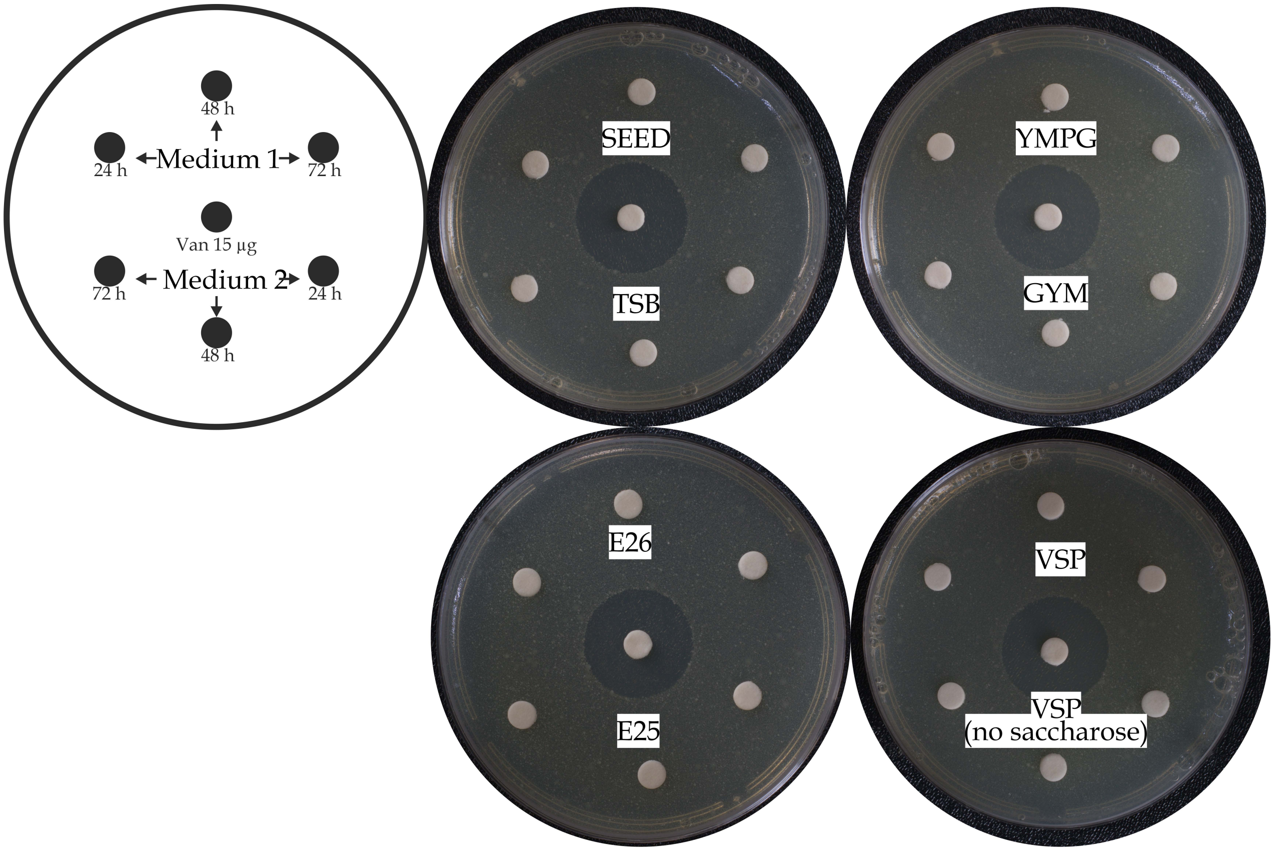

(b)

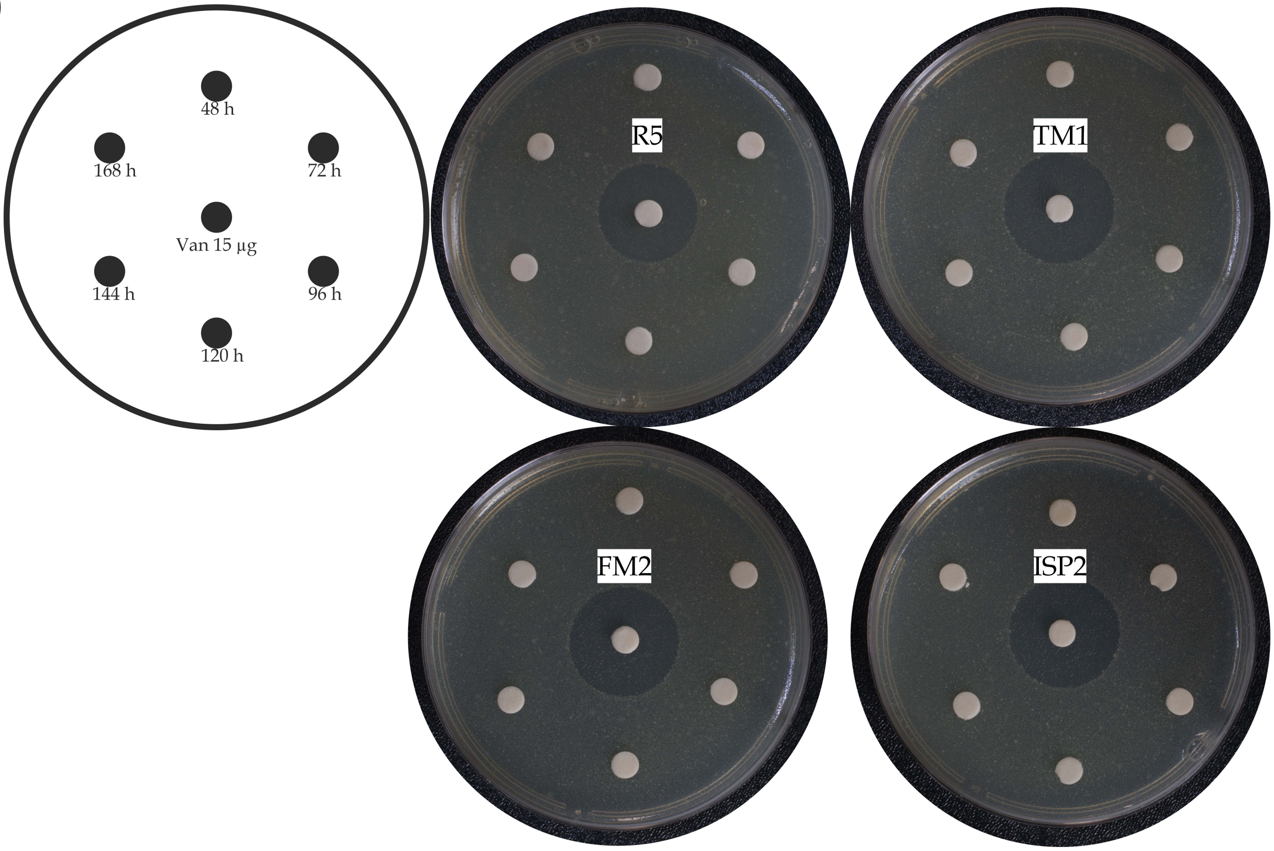

Figure S20. No GPA-related antimicrobial activities were observed in the samples of supernatant of submerged *A. bartoniae* DSM 45807 cultivated in vegetative **(a)** or GPA production media **(b)** at all tested time points.

Supplementary References

1. Kieser, T.; Bibb, M.; Buttner, M.; Chater, K. *Practical Streptomyces Genetics. 2nd ed.*; 2000; ISBN 0-7084-0623-8.

2. Taurino, C.; Frattini, L.; Marcone, G.L.; Gastaldo, L.; Marinelli, F. *Actinoplanes teichomyceticus* ATCC 31121 as a cell factory for producing teicoplanin. *Microb. Cell Fact.* **2011**, *10*, doi:10.1186/1475-2859-10-82.

3. Yushchuk, O.; Ostash, I.; Mösker, E.; Vlasiuk, I.; Deneka, M.; Rückert, C.; Busche, T.; Fedorenko, V.; Kalinowski, J.; Süssmuth, R.D.; et al. Eliciting the silent lucensomycin biosynthetic pathway in *Streptomyces cyanogenus* S136 via manipulation of the global regulatory gene *adpA*. *Sci. Rep.* **2021**, *11*, doi:10.1038/s41598-021-82934-6.

4. Jung, H.M.; Kim, S.Y.; Prabhu, P.; Moon, H.J.; Kim, I.W.; Lee, J.K. Optimization of culture conditions and scale-up to plant scales for teicoplanin production by *Actinoplanes teichomyceticus*. *Appl. Microbiol. Biotechnol.* **2008**, *80*, 21–27, doi:10.1007/s00253-008-1530-2.

5. Marcone, G.L.; Binda, E.; Carrano, L.; Bibb, M.; Marinelli, F. Relationship between glycopeptide production and resistance in the actinomycete *Nonomuraea* sp. ATCC 39727. *Antimicrob. Agents Chemother.* **2014**, *58*, 5191–5201, doi:10.1128/AAC.02626-14.

6. Koshla, O.; Yushchuk, O.; Ostash, I.; Dacyuk, Y.; Myronovskyi, M.; Jäger, G.; Süssmuth, R.D.; Luzhetskyy, A.; Byström, A.; Kirsebom, L.A.; et al. Gene *miaA* for post-transcriptional modification of $tRNA_{XXA}$ is important for morphological and metabolic differentiation in *Streptomyces*. *Mol. Microbiol.* **2019**, *112*, 249–265, doi:10.1111/mmi.14266.

7. Blin, K.; Shaw, S.; Augustijn, H.E.; Reitz, Z.L.; Biermann, F.; Alanjary, M.; Fetter, A.; Terlouw, B.R.; Metcalf, W.W.; Helfrich, E.J.N.; et al. AntiSMASH 7.0: New and improved predictions for detection, regulation, chemical structures and visualisation. *Nucleic Acids Res.* **2023**, *51*, W46–W50, doi:10.1093/nar/gkad344.

8. Sievers, F.; Higgins, D.G. Clustal Omega. *Curr. Protoc. Bioinforma.* **2014**, *2014*, 3.13.1-3.13.16, doi:10.1002/0471250953.bi0313s48.

9. Jones, D.T.; Taylor, W.R.; Thornton, J.M. The rapid generation of mutation data matrices from protein sequences. *Bioinformatics* **1992**, *8*, 275–282, doi:10.1093/bioinformatics/8.3.275.

10. Whelan, S.; Goldman, N. A general empirical model of protein evolution derived from multiple protein families using a maximum-likelihood approach. *Mol. Biol. Evol.* **2001**, *18*, 691–699, doi:10.1093/oxfordjournals.molbev.a003851.

11. Le, S.Q.; Gascuel, O. An improved general amino acid replacement matrix. *Mol. Biol. Evol.* **2008**, *25*, 1307–1320, doi:10.1093/molbev/msn067.
